# A systematic review on gut microbiota in type 2 diabetes mellitus

**DOI:** 10.3389/fendo.2024.1486793

**Published:** 2025-01-17

**Authors:** Serena Chong, Mike Lin, Deborah Chong, Slade Jensen, Namson S. Lau

**Affiliations:** ^1^ South West Sydney Limb Preservation and Wound Research, Ingham Institute for Applied Medical Research, Sydney, NSW, Australia; ^2^ South West Clinical School, Faculty of Medicine, University of New South Wales, Sydney, NSW, Australia; ^3^ Department of Endocrinology, Royal Prince Alfred Hospital, Sydney, NSW, Australia; ^4^ Garvan Institute of Research, Sydney, NSW, Australia; ^5^ Animal Health Laboratory, Department of Natural Resources and Environment Tasmania, Tasmania, TAS, Australia; ^6^ Infectious Disease and Microbiology, Ingham Institute for Applied Medical Research, Sydney, NSW, Australia; ^7^ School of Medicine Antibiotic Resistance and Mobile Elements Groups, Ingham Institute for Applied Medical Research, Sydney, NSW, Australia; ^8^ Liverpool Diabetes Collaboration, Ingham Institute of Applied Medical Research, Sydney, NSW, Australia

**Keywords:** gut microbiota, diabetes, gut dysbiosis, diabetes mellitus, systematic review

## Abstract

**Aims/hypothesis:**

The gut microbiota play crucial roles in the digestion and degradation of nutrients, synthesis of biological agents, development of the immune system, and maintenance of gastrointestinal integrity. Gut dysbiosis is thought to be associated with type 2 diabetes mellitus (T2DM), one of the world’s fastest growing diseases. The aim of this systematic review is to identify differences in the composition and diversity of the gut microbiota in individuals with T2DM.

**Methods:**

A systematic search was conducted to identify studies reporting on the difference in gut microbiota composition between individuals with T2DM and healthy controls. Relevant studies were evaluated, and their characteristics and results were extracted using a standardized data extraction form. The studies were assessed for risk of bias and their findings were reported narratively.

**Results:**

58 observational studies published between 2010 and 2024 were included. Beta diversity was commonly reported to be different between individuals with T2DM and healthy individuals. Genera Lactobacillus, Escherichia-Shigella, Enterococcus, Subdoligranulum and Fusobacteria were found to be positively associated; while Akkermansia, Bifidobacterium, Bacteroides, Roseburia, Faecalibacteirum and Prevotella were found to be negatively associated with T2DM.

**Conclusions:**

This systematic review demonstrates a strong association between T2DM and gut dysbiosis, as evidenced by differential microbial abundances and altered diversity indices. Among these taxa, *Escherichia-Shigella* is consistently associated with T2DM, whereas *Faecalibacterium prausnitzii* appears to offer a protective effect against T2DM. However, the heterogeneity and observational nature of these studies preclude the establishment of causative relationships. Future research should incorporate age, diet and medication-matched controls, and include functional analysis of these gut microbes.

**Systematic review registration:**

https://www.crd.york.ac.uk/prospero/, identifier CRD42023459937.

## 1 Introduction

The human body hosts a vast population of microorganisms, including archaebacteria, viruses, fungi and eubacteria (also referred to as bacteria), collectively referred to as microbiota. The period of initial gut colonization in humans remains a contentious topic, with some studies suggesting such colonization occurs *in utero*, while others refute this suggestion. Regardless, it is widely accepted that in humans, the infant gut microbiota is rapidly populated near the time of birth, typically achieving stability between the ages of 2 and 5 (1).

Due to factors such as peristalsis, pH, oxygen and biological products, the microbiota varies throughout different parts of the gastrointestinal tract. The small intestine contains fewer microorganisms due to a faster transit time, acidic environment, and the presence of bile and pancreatic secretions. In contrast, the large intestine hosts billions of microorganisms, mainly dominated by anaerobic bacteria, including Firmicutes, Bacteroides, Actinobacteria, Proteobacteria and Verrucomicrobia (2). This is the primary site where the microbiota interact with the human host (3).

Gut microbiota are involved in core human bodily functions including digestion and nutrient degradation, synthesis of biological agents, immune system development and maintenance of gut integrity (4). Significant factors that influence the microbiotia gut composition include age, gender, geographical location and diet. Additionally, prebiotics and probiotics have been used to change the composition of gut microbiota and induce beneficial effects. It has also been suggested that early microbial transfer during the formation and development of the gut microbiota may play a role in the inheritability of human conditions such as neurological diseases and obesity (5).

The gut bacterial microbiome has been associated with the pathophysiology of multiple chronic diseases, one of which is Type 2 diabetes mellitus (T2DM) (6–8). Type 2 diabetes mellitus (T2DM) is characterized by chronic hyperglycemia due to decreased insulin secretion by pancreatic beta cells and increased insulin resistance. Rapid urbanization, nutrition transition and sedentary lifestyles have led to a drastic rise in cases (9). In 2018 there were over 500 million cases of T2DM globally (172). In Australia, the number of patients with T2DM increased to 1 million accounting for 2.3% ($2.7 billion AUD) of total disease expenditure in 2015-2016.

Increasing evidence shows that alterations in gut bacterial microbiota plays a crucial role in the development of T2DM. Gut bacterial dysbiosis in individuals with T2DM is thought to cause systemic inflammation and altered metabolism, leading to increased peripheral insulin resistance (4). Over time, this can lead to the development of complications such as diabetes related foot complications. Hence, it is crucial to identify bacteria contributing to the development and exacerbation of this disease, as well as those that play a protective role in preventing it.

## 2 Aim of systematic review

Several studies have established that the composition and function of gut bacterial microbiota in individuals with T2DM are different from healthy individuals. Despite this, the specific microbial changes remain largely unknown. This systematic review aims to provide an updated review on whether the gut bacterial microbiota profile of individuals with T2DM differ from healthy individuals. Mechanisms contributing to the pathophysiology of T2DM will also be discussed.

## 3 Methods

### 3.1 Search strategy

We performed a detailed systematic review of published data according to the PRISMA (Preferred Reporting Items for Systematic Reviews and Meta Analyses) guidelines. The methodological approach was registered in PROSPERO (International prospective register of systematic reviews) database under protocol number CRD42023459937.

Embase and PubMed literature search was performed on articles between Jan 1^st^ 2010 and April 15^th^ 2024. The search strategy combined MESH (Medline) and free terms using the boolean operators “AND” and “OR”. “Diabetes Mellitus”, “gut microbiome”, “intestinal flora” and “gastrointestinal microbiome” were terms used in the search. A complementary search was carried out in the references of studies included. The search protocol is shown below:

((“Diabetes Mellitus”[Majr: NoExp] OR “Diabetes Mellitus, Type 2”[Majr: NoExp] OR T2D[Text Word] OR type 2 diabetes[Text Word] OR “type 2 diabetes mellitus”[Title/Abstract:~2]) AND (“Gastrointestinal Microbiome”[Majr] OR gut micro*[Text Word] OR intestine flora[Text Word] OR intestinal flora[Text Word] OR gut flora[Text Word] OR intestine micro*[Text Word] OR intestinal micro*[Text Word] OR Gastrointestinal micro*[Text Word])) NOT (animals[Mesh] NOT humans[Mesh])

### 3.2 Eligibility criteria

All original peer reviewed research publications were considered. Eligible studies included observational human studies specifically examining gut microbiota in T2DM patients compared with control groups.

Exclusions: studies on type 1 diabetes mellitus or gestational diabetes; those without control groups; longitudinal studies; studies on children or adolescents aged <18 years or in the elderly aged >80 years; non-English studies; studies with only abstracts available; and studies with high risk of bias.

Microbial taxa were defined as positively or negatively associated with T2DM if p value <0.05 when comparing taxa abundance between individuals with T2DM and healthy controls. For linear discriminant analysis (LDA), a score of >4 indicated a positively association, while <4 indicated a negative association. For prospective studies with interventions, the baseline result was used. For studies with more than one population group, results were only reported to be positively or negatively associated if both groups demonstrated the result. Microbial taxa without reported p values, p values >0.05 or LDA values <4 were classified as non-significant and into an increased, decreased or equivocal (equal abundance or not reported) trend.

The titles and abstracts of all identified studies were reviewed by two independent authors. Studies were assessed using the Newcastle–Ottawa Quality Assessment Scale. This instrument included three domains: selection, comparability, and outcomes. High risk of bias was determined when some of the domains did not receive a point, in which case that study was excluded. Ambiguities in selection criteria were resolved by discussions between at least 3 researchers.

### 3.3 Data extraction

The data extracted from the studies included in this systematic review are summarized in **Supplementary Table 1** with the following information: author and year of publication, country and period of study/seasons (if available), sample size and characterization of the study population, method used to evaluate the gut microbiota and bacteria analyzed (if applicable), and outcomes.

## 4 Results and discussion

**Figure 1:**
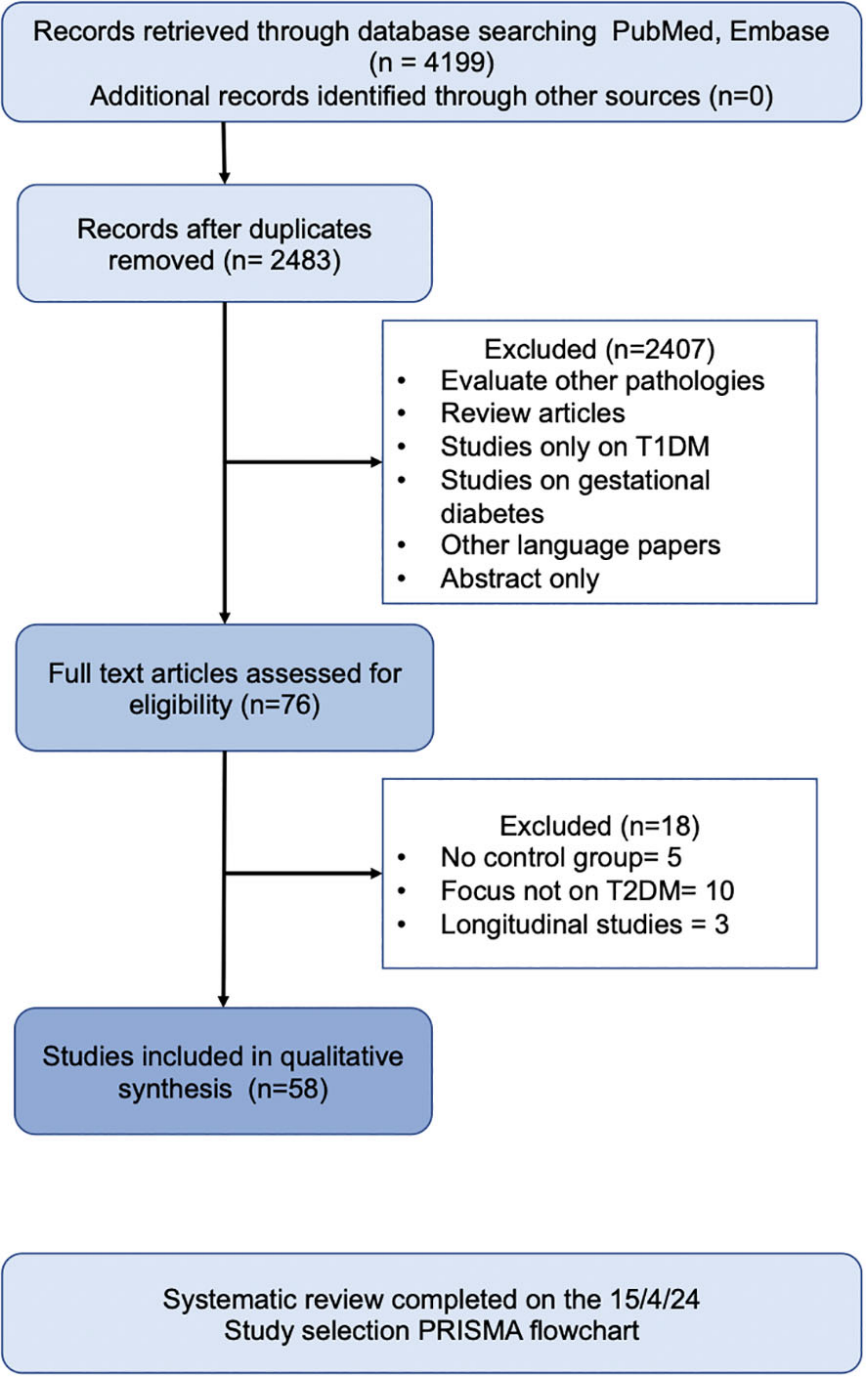
Search Strategy.

In total, 58 human observational studies were included in this review (**Figure 1**). The majority of these studies reported associations between specific taxa and the development and exacerbation of T2DM. However, no taxa were universally agreed upon to be positively or negatively associated with T2DM.

### 4.1 Alpha and beta diversity

#### 4.1.1 Alpha diversity

Alpha diversity refers to the microbial species diversity (richness) within a functional community. Reported indices included the Shannon index, Chao1 index, Simpson index, Faith index, Observed index, Abundance-based Coverage Estimator (ACE) index and Good’s Coverage. The Shannon index was the most commonly reported metric. A p value of <0.05 was deemed statistically significant. Most analyses reported no difference in alpha diversity between T2DM individuals and healthy controls (**Table 1**). Alpha diversity metrics varied by ethnicity, oral antihyperglycemic agents and other environmental factors (20, 31, 40). Higher diversity was observed in treatment naïve T2DM individuals compared to those receiving treatment (41).

**Table 1:** PMID of studies reporting alpha diversity indices in type 2 diabetes compared to controls.

Alpha diversity Indices	Increased in T2DM	Reduced in T2DM	No significant difference
Shannon	(10, 11)	(12–18)	(19–33)
Chao 1	(10)	(14, 18, 27, 34, 35)	(13, 21, 25, 26, 30, 31, 36, 37)
Simpson		(12)	(25–28, 31)
Faith	(10)	(13, 34)	(21, 22, 36)
Observed	(10, 11)	(16, 21)	(20, 22, 25, 26, 29, 31, 36, 38)
ACE		(27)	(25, 26, 29, 31)
Good’s Coverage		(36)	(25)
Unspecified			(39)

#### 4.1.2 Beta diversity

Beta diversity describes the amount of differentiation and dissimilarities between gut bacterial microbiota communities. The most common beta diversity metric used was the unweighted Unifrac distance. A p value of < 0.05 was deemed significant. The majority of studies reported a significant difference in beta diversity in individuals with T2DM compared to healthy controls (**Table 2**).

**Table 2:** Summary of studies reporting beta diversity in type 2 diabetes compared to controls.

	Significant difference	Difference	No significant difference
Beta Diversity	(10, 13–17, 22, 27, 34–36, 39, 41)	(21, 25, 37)	(11, 19, 23, 24, 26, 29, 30, 38, 42)

If difference in beta diversity was observed but no p values were reported, they were classified as having difference.

### 4.2 Phylum analysis - prevalence of firmicutes, bacteroidetes and the firmicutes/bacteroidetes ratios

**Figure 2:**
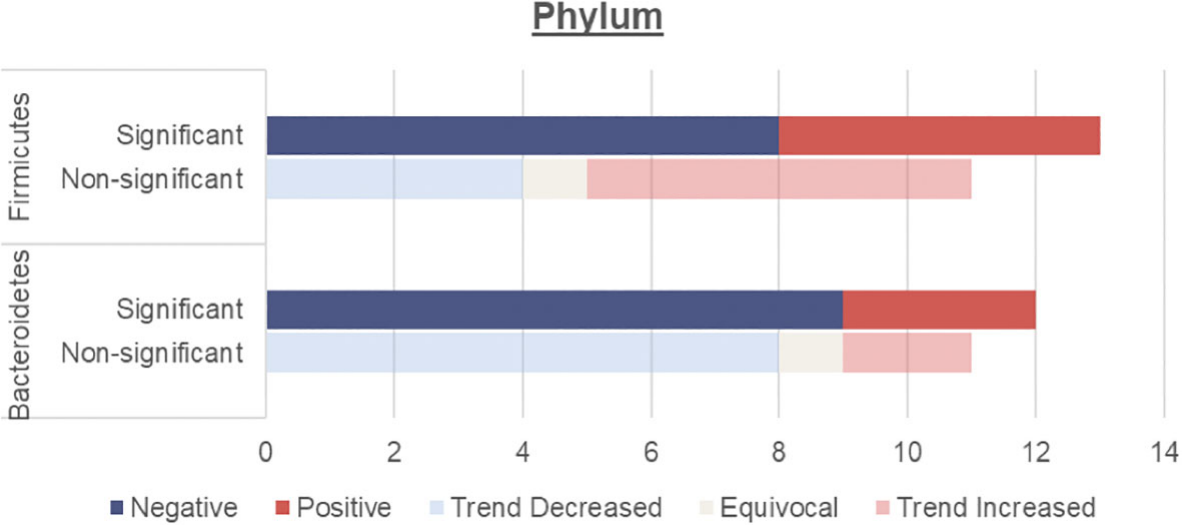
Number of human studies reporting Firmicutes and Bacteroidetes abundance and their association with T2DM. Studies were classified as having a significant association with T2DM (either positive or negative) if the p values were <0.05. Studies were classified as having a non-significant association with T2DM if they did not report on p values, had p values >0.05 or an LDA value <4 or >-4. These studies were then further classified into a non-significant association but trend increased, equivocal (equal abundance or not reported), or trend decreased.

This review focuses on the phylum and genus levels of gut bacteria. The human gut bacterial microbiota consists mainly of Firmicutes and Bacteroidetes, which make up over 90% of the community. The remaining 10% includes phyla like Proteobacteria, Actinobacteria and Verrucomicrobia. In individuals with T2DM, the most commonly altered phyla are Firmicutes and Bacteroidetes (**Figure 2**, **Table 3**).

**Table 3:** Summary of studies reporting Firmicutes and Bacteroidetes abundance.

	Increase	Decrease	No significant difference
Firmicutes	(18, 21, 34, 36, 41)	(11, 13, 35, 37, 43–46)	↑ (19, 25, 26, 30, 33, 38) ↓ (14, 16, 24, 39) Equivocal (47)
Bacteroidetes	(11, 35, 44)	(12, 16, 18, 34, 36, 38, 39, 41, 48)	↑ (14, 37) ↓ (13, 19, 21, 25, 26, 30, 33, 43) Equivocal (24)

Studies with no significant differences are reported as trends. ↑ - increase, ↓ - decrease.

#### 4.2.1 Firmicutes and bacteroidetes

Overall, an unchanged Firmicutes and reduced Bacteroidetes abundance were observed among individuals with T2DM.

An unchanged Firmicutes abundance may be due to a simultaneous increase in opportunistic Firmicutes pathogens such as *Enterococcus* (**Table 4**), *Eisenbergiella* (16) *Acidaminococcus* (29, 41) and a decrease in beneficial Firmicutes microbes including *Faecalibacterium* and *Roseburia* (**Table 5**)

**Table 4:** Genera found to be positively associated with type 2 diabetes.

Genus	Increased	Decreased	No significant difference
*Lactobacillus*	(15–17, 21, 33, 49–54)	(55)	↑ (12, 25, 26, 36, 47) ↓ (14) Equivocal (19, 41)
*Escherichia-Shigella*	(12, 13, 16, 18, 38, 40, 47)	(36)	↑ (25, 41, 56) ↓ (55)
*Subdoligranulum*	(12, 30, 32, 38)	(20, 55)	↑ (26, 36)
*Enterococcus*	(12, 16, 27)		↑ (26) ↓ (10, 50) Equivocal (51)
*Fusobacterium*	(16, 26, 34)		↑ (14, 25, 54) ↓ (54, 55)

Studies with no significant differences are reported as trends. ↑ - increase, ↓ - decrease.

**Table 5:** Genera found to be negatively associated with type 2 diabetes.

Genus/Species	Increased	Decreased	No significant difference
Akkermansia	(12)	(34)	↑ (14, 33, 39) ↓ (55) Equivocal (41)
*Akkermansia Muciniphila*	(32, 57)	(23, 30, 35, 43, 44, 49)	↑ (58)
Bifidobacterium	(12, 15, 16, 18, 26, 59)	(10, 17, 34, 47, 49, 52, 54, 60)	↑ (14, 61) ↓ (25, 39, 50, 55) Equivocal (19, 33, 51)
*Bacteroides*		(12, 17, 25, 38, 39)	↑ (14, 61) ↓ (26, 47, 55, 59). Equivocal (19, 30, 35).
*Roseburia*	(10)	(16, 27, 30, 39, 55, 59)	↑ (34, 61) ↓ (12, 14, 17, 18, 26) Equivocal (19)
*Faecalibacterium*	(10, 16)	(12, 15, 17, 18, 26, 32, 34, 35)	↑ (34, 61) ↓ (14, 39, 40, 47, 55, 59)
*Faecalibacterium prausnitzii*		(15, 21, 30, 32, 43, 44, 46, 49, 57, 62)	↓ (14, 23, 40) Equivocal (58)
*Prevotella*	(10, 11)	(15, 16, 36, 38, 51)	↑ (30, 35, 47, 54, 59) ↓ (12, 14, 17, 34, 41, 50, 55, 61) Equivocal (33)

Studies with no significant differences are reported as trends. ↑ - increase, ↓ - decrease.

Meanwhile, Bacteroidetes are thought to be beneficial to human health with several genera including *Bacteroides* and *Prevotella* considered an untapped resource for next-generation prebiotics. Both these taxa, proposed to mitigate metabolic endotoxaemia and inflammation, were reduced among individuals with T2DM (**Table 5**). Bacteroidetes have negative correlation with fasting blood glucose levels (27, 36), corresponding with their reduced levels in T2DM.

#### 4.2.2 The firmicutes/bacteroidetes ratio

The Firmicutes/Bacteroidetes (F/B) ratio (**Table 6**) represents the relationship between two dominant phyla and is commonly used as a marker of gut dysbiosis.

**Table 6:** Firmicutes-Bacteroides Ratio.

	Increased	Suggestive reduced	Suggestive increased
Firmicutes/Bacteroides Ratio	(27, 38, 39, 41)	(14, 26, 37, 43)	(33, 48, 58)

Considered suggestive if no significance was reported or if p >0.05.

The F/B ratio was not consistently associated with clinical parameters. Larsen et al. found a positive correlation between the Bacteroidetes to Firmicutes ratio and plasma glucose (37) while Wang et al. reported a positive correlation between the F/B ratio and body mass index (BMI), fasting blood glucose levels and HBA1c (27). Other studies found no correlation with fasting, postprandial blood glucose levels (30), age, HBA1c or lipid profile (39). This suggests that while the F/B ratio indicates dysbiosis, it does not specifically predict metabolic outcomes.

### 4.3 Genera analysis - bacteria involved in type 2 diabetes

#### 4.3.1 Genera of bacteria found to be increased in individuals with type 2 diabetes

**Figure 3:**
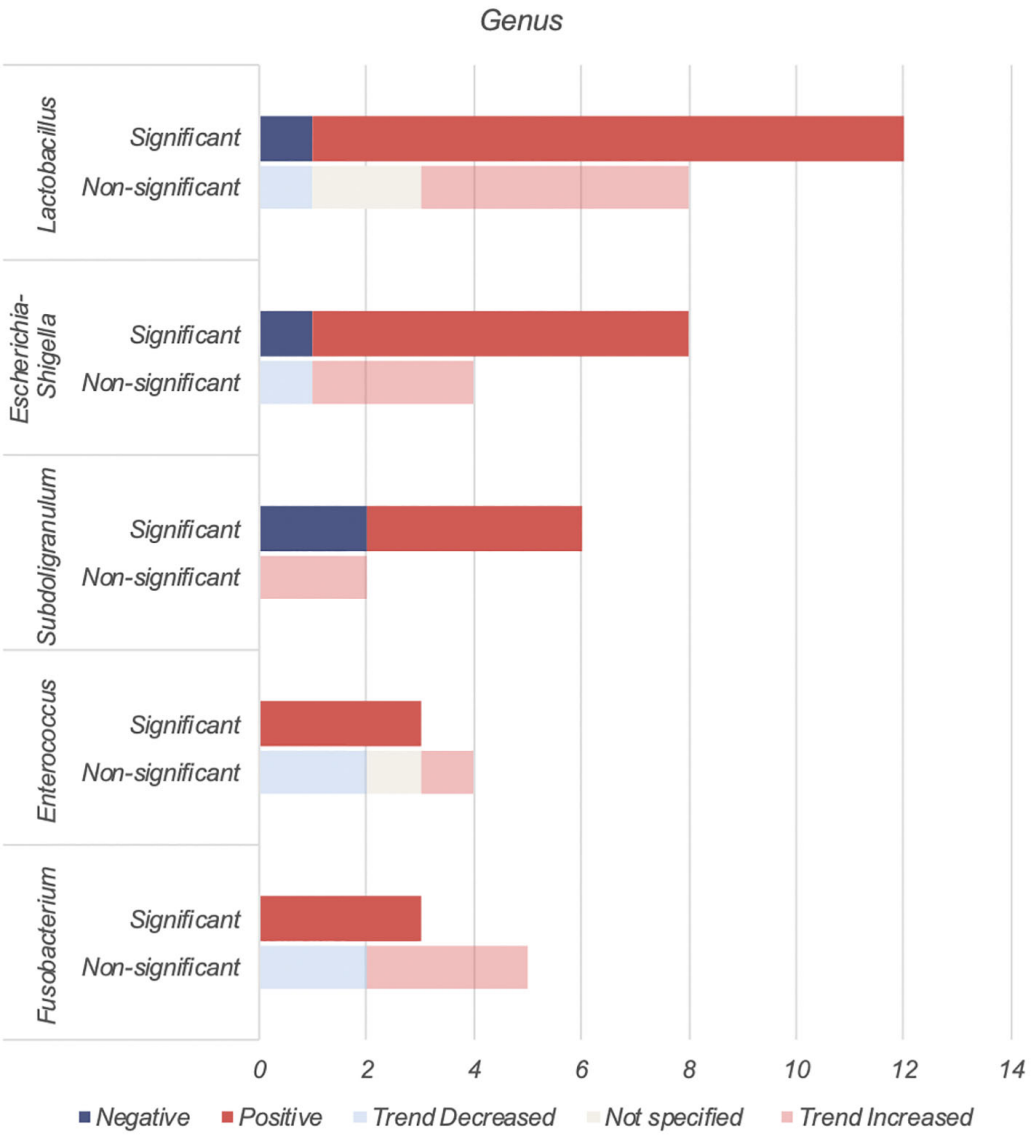
Number of human studies reporting on genera found to be positively associated with T2DM.

*Lactobacillus*, *Escherichia-Shigella*, *Enterococcus*, *Subdoligranulum* and *Fusobacteria* were found to be positively associated with T2DM (**Table 4**, **Figure 3**).

##### 4.3.1.1 Lactobacillus

The Lactobacillus genus comprises of over 200 physiologically diverse gram-positive, non-spore forming lactic acid bacteria. Despite its positive association with T2DM, *Lactobacillus* species such as *Lactobacillus paracasei* (63)*, Lactobacillus fermentum* (64) *Lactobacillus acidophilus* and *Lactobacillus rhamnosus* (65, 66) have demonstrated anti-inflammatory properties or benefits on host metabolism as a combination probiotic with *Bifidobacterium lactic* (65, 66).

The positive association of *Lactobacillus* with T2DM may therefore be driven by Metformin. Metformin, a first-line antihyperglycemic agent for treatment of T2DM, may alter bacterial abundances depending on the taxon’s resistance or sensitivity to the drug. In 2015, using 784 human gut metagnomes, Forslund et al. confirmed this positive association between metformin and Lactobacillus (55).

Among the eleven studies that reported an increase in *Lactobacillus* abundance (15–17, 21, 33, 49–54), only three studies (21, 50, 52) accounted for metformin use. Among these, one study found higher *Lactobacillus* levels regardless of metformin use (50), one found higher levels only in participants on unspecified oral antihyperglycemic agents (21), while the last study found no difference when accounting for metformin (52). More studies on treatment naïve T2DM or controlled for Metformin use are warranted.

##### 4.3.1.2 Escherichia-Shigella

The *Escherichia-Shigella* genus, part of the family Enterobacteriaceae, includes multiple opportunistic pathogens (67). These gram-negative bacteria produce proinflammatory components such as lipopolysaccharide (LPS) and peptidoglycans, leading to intestinal and systemic inflammation (12). This systemic inflammation and consequent insulin resistance are key drivers for T2DM.

Unsurprisingly, *Escherichia-Shigella* abundance correlates with variables related to diabetes and obesity, including insulin resistance, diminished beta cell function (56), fasting glucose (41), HBA1c and BMI (47). This genus has been implicated in T2DM complications such as peripheral neuropathy (68), autonomic neuropathy (69), retinopathy (70), diabetic nephropathy (71) and chronic diabetic foot infections (72). *Escherichia-Shigella* has also been associated with an increasing abundance from healthy controls, pre-diabetes to T2DM (56). An increase *in Escherichia-Shigella* has also been associated with metformin use (13, 73). The outlier study that reported decreased *Escherichia-Shigella* abundance may be due to dietary or environmental differences (36).

##### 4.3.1.3 Subdoligranulum

*Subdoligranulum* are anaerobic, spore-free gram-negative bacteria (12). This genera remains relatively underexplored and has only two known species - *Subdoligranulum variabile* and *Subdoligranulum didolesgii*. Four studies (12, 30, 32, 38) found Subdoligranulum more common in T2DM (**Table 4**) while two studies reported a negative association between T2DM and *Subdoligranulum variabile* (46, 74). These discrepancies may be related to species-specific properties.

*Subdoligranulum* has been linked to both promotion (75) and reduction of chronic inflammation (74). *Subdoligranulum didolesgii* has been associated with rheumatoid arthritis by triggering synovitis, while *Subdoligranulum variabile* has anti-inflammatory properties through short chain fatty acid (SCFA) production. Decreased levels of *Subdoligranulum variabile* in T2DM individuals may be suggestive of an overall state of inflammation (46).

*Subdoligranulum’s* positive association with T2DM may be influenced by metformin use (55). Of four studies reporting increased *Subdoligranulum*, two did not report metformin use (12, 32), one excluded metformin users (30), and one found an increase regardless of metformin use (38).

##### 4.3.1.4 Enterococcus

*Enterococcus* are gram-positive facultative anaerobic cocci found in intestinal microbiota and on the skin. Some species are opportunistic pathogens causing severe infections such as bacterial endocarditis and spontaneous bacterial peritonitis, while others (*Enterococcus durans*) produce anti-inflammatory SCFAs (76).

*Enterococcus* may contribute to the development of T2DM through two mechanisms. Firstly, *Enterococcus faecalis* secretes matrix metalloprotease gelatinase causing chronic intestinal inflammation and impaired gut barrier integrity (77), leading to systemic inflammation. Secondly, *Enterococcus* has been linked to impaired glucose homeostasis. Associations include higher HBA1c (16, 27), fasting (27) and post prandial (16) glucose levels, and impaired beta cell function (27). Mechanistically this may relate to overgrowth of enterococcus leading to proportional decreases in beneficial anti-inflammatory bacteria (50).

##### 4.3.1.5 Fusobacterium

*Fusobacterium* are anaerobic gram-negative rod bacteria. Similar to *Enterococcus*, this genus is part of the regular colorectal microbiota. *Fusobacterium*, in particular *Fusobacterium nucleatum*, has been associated with increased production of inflammatory cytokines such as IL-6, IL-8, TNF-α and COX-2 (78). This may contribute to the chronic inflammatory state seen in T2DM. *Fusobacterium* has also been associated with diabetic nephropathy (79) and its species found increased among individuals with T2DM (23, 44).

#### 4.3.2 Genera of bacteria found to be reduced in individuals with type 2 diabetes

**Figure 4:**
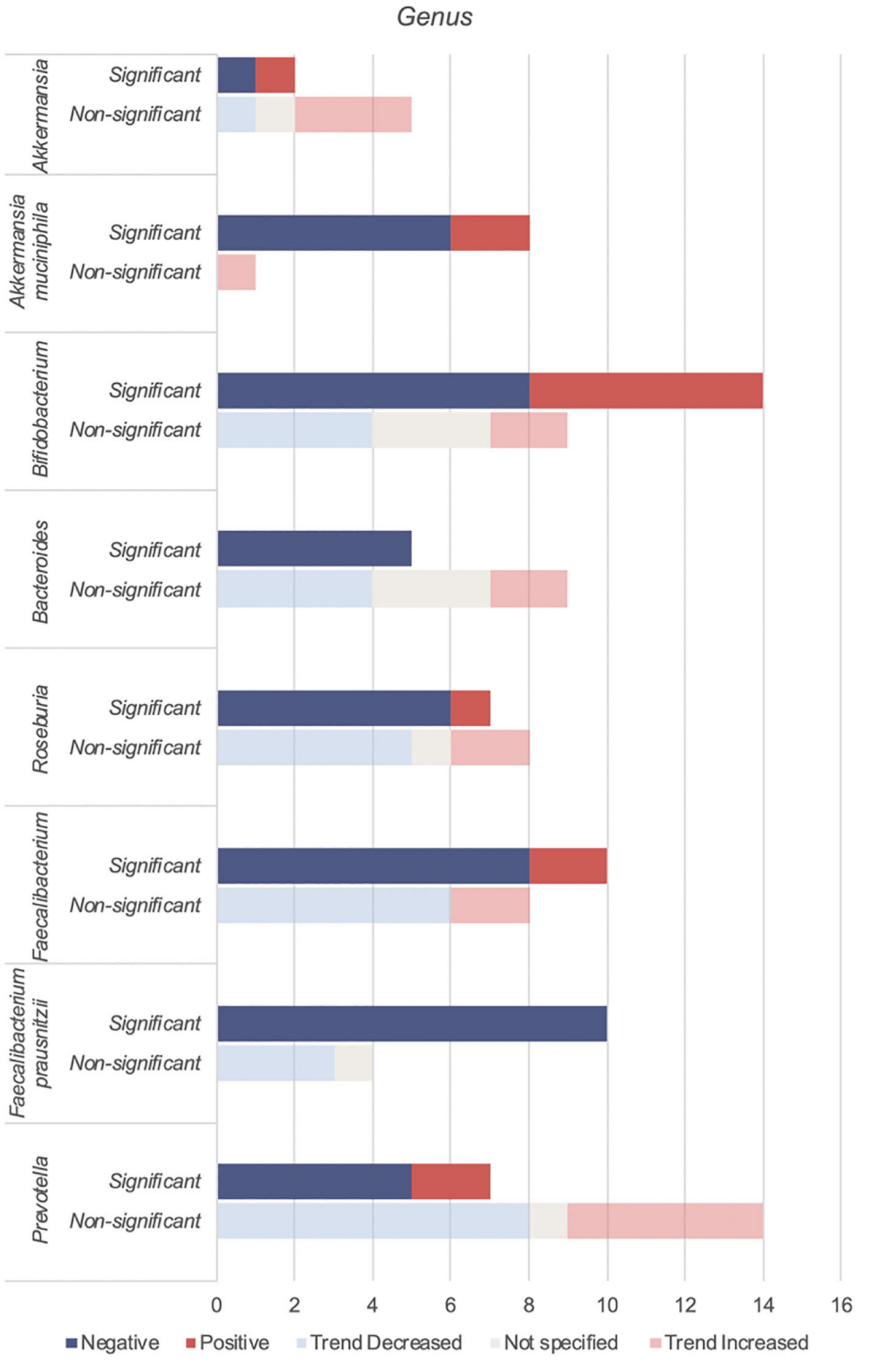
Number of studies reporting on genera found to be negatively associated with T2DM.

*Akkermansia*, *Bifidobacterium*, *Bacteroides*, *Roseburia*, *Faecalibacteirum* and *Prevotella* were found to be negatively associated with T2DM (**Table 5**, **Figure 4**). Species abundance of *Bifidobacterium*, *Bacteroides*, *Roseburia* and *Prevotella* can be found in **Supplementary Table 2.**

##### 4.3.2.1 Akkermansia

*Akkermansia* is gram-negative bacterium belonging to the Verrucomicrobia phylum. *Akkermansia mucinphilia*, a symbiont microbe colonizing the human intestinal mucosal barrier, is a promising next generation probiotic. It plays a critical role in the maintenance of intestinal barrier, production of anti-inflammatory cytokines and SCFA benefiting host metabolism. In diabetic rat models, administration of live attenuated *Akkermansia* reduced oxidative stress, lipotoxicity, LPS and inflammation (80). In individuals with T2DM, combined probiotics containing *Akkermansia muciniphila* reduced HBA1c and postprandial glucose control (81).

Reduced levels of *Akkermansia mucinphilia* are associated with T2DM. *Akkermansia* is inversely correlated with HBA1c and fasting glucose and positively with anti-oxidants (41).

##### 4.3.2.2 Bifidobacterium

*Bifidobacterium* is a dominant non-spore-forming, gram-positive taxa that help maintain balances between the various intestinal floras (82). Key *Bifidobacterium* species include *Bifidobacteroim bifidum*, *Bifidobacterium adolescentis* and *Bifidobacterium longum*. These species have been used as probiotics in humans (65, 66, 83) and administered in animal studies (84, 85) leading to reduced cytokine production and improved metabolic parameters such as glucose and HBA1c (66, 84).

Apart from SCFA production, *in vivo* and *in vitro* studies show that *Bifidobacterium* administration markedly decreased intestinal permeability by increasing tight junction expression and reducing inflammatory cytokines such as IL-6 and TNF-α (86). This reduces metabolic endotoxaemia, systemic inflammation and may explain its overall negative association with T2DM (**Table 5**). An increase in *Bifidobacterium* has been attributed to antihyperglycemic agents (16) or a U shaped association with T2DM (26, 59).

##### 4.3.2.3 Bacteroides

*Bacteroides* is a gram-negative obligate anaerobic taxa constituting approximately 25% of the intestinal gut microbiota. As commensals, these taxa generally maintain a beneficial relationship with the human gut. Overall, *Bacteroides* species including *Bacteroides fragilis*, *Bacteroides thetaiotamicron*, *Bacteroides vulgutas* or *Bacteroides dorei* have been associated with a protective effect against T2DM through anti-inflammatory properties (87) and an improved gut barrier integrity from mucus (88) and SCFA production (89). *Bacteroides* species also have a structurally different LPS that is less pro-inflammatory than classical enterobacterial LPS (90). Discrepancies in *Bacteroides* abundance (**Table 5**) may be due to the bacteriostatic and bactericidal effect of metformin (55) or potential pathogenic Bacteroides species that can contribute to chronic inflammation (39).

##### 4.3.2.4 Roseburia

*Roseburia* is a gram-positive, SCFA-producing member of the Firmicutes phylum that inhabits the human colon. *Roseburia* has been identified as a pathognomonic bacteria in T2DM (91) with significant lower levels in participants. Reduced species include *Roseburia hominis* (23, 46), *Roseburia intestinalis* and *Roseburia inulinivorans* (32, 53, 55). *Roseburia* improves glucose homeostasis and intestinal permeability through SCFA production and anti-inflammatory properties (92). Gut microbiota transplantations from lean donors to recipients with metabolic syndrome led to increased fecal Roseburia and butyrate levels, correlating with improved insulin sensitivity (93).

##### 4.3.2.5 Faecalibacterium

*Faecalibacterium* are human gut colonizers and well-known SCFA producers. *Faecalibacterium* and *Faecalibacterium prausnitzii* were consistently reduced in T2DM (**Table 5**), with the later being highly discriminant (91). In mice, *Faecalibacterium prausnitzii* administration was associated with improved glucose levels and HBA1c, making it a promising orally administered probiotic (94). *Faecalibacterium* is negatively associated with HBA1c (39).

##### 4.3.2.6 Prevotella

*Prevotella* has been linked to both pathogenic effects including systemic inflammation and insulin resistance (95) and beneficial effects like SCFA production (96) and reduced gut permeability via increased production of tight junction proteins (97). *Prevotella* is negatively correlated with HBA1c (16, 41, 98), but positively with blood glucose (10, 41). The discrepancies within the *Prevotella* genus may be due to diet (24) and genetic diversity within its species (99).

#### 4.3.3 Genera of bacteria found to have mixed findings in type 2 diabetes

Unlike previous reviews (100), *Blautia* and *Ruminococcus* were found to have mixed associations (**Table 7**).

**Table 7:** Genera found to have mixed associations with type 2 diabetes.

Genus	Increased	Decreased	No significant difference
*Blautia*	(15, 18, 26, 27, 101)	(12, 35, 46, 59, 61)	↑ (38, 39) ↓ (28, 55, 56) Equivocal (30, 41)
*Ruminococcus*	(17, 30, 39)	(11, 27, 36)	↑ (18, 22, 34, 47) ↓ (10, 12, 14, 28, 38, 40, 55) Equivocal (28, 41, 59, 61)

Studies with no significant differences are reported as trends. ↑ - increase, ↓ - decrease.

### 4.4 Microbiota effects on metabolism in type 2 diabetes individuals

**Figure 5:**
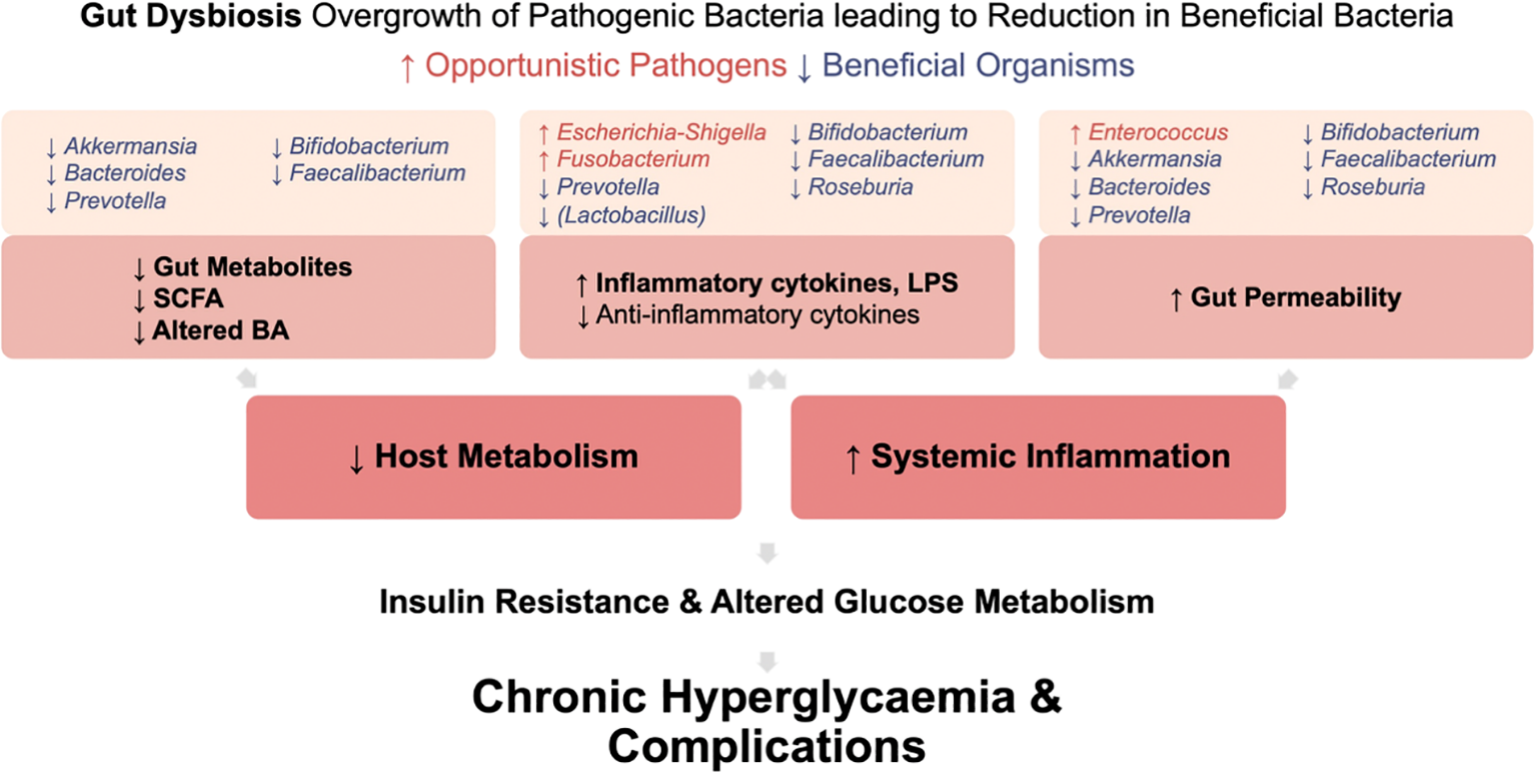
Mechanisms by which gut dysbiosis contributes to the development and progression of T2DM. Gut dysbiosis in T2DM leads to increased systemic inflammation and an unfavorable host metabolism. This occurs due to increased production of pro-inflammatory cytokine and LPS, increased gut permeability enabling bacterial endotoxin translocation, and reduced production of beneficial gut metabolites. Ultimately, this systemic inflammation induces insulin resistance. Coupled with altered glucose metabolism in T2DM, these factors contribute to chronic hyperglycemia and the development of complications.

In T2DM, gut dysbiosis leads to increased systemic inflammation and an unfavorable host metabolism (**Figure 5**). This is due to an increase in pro-inflammatory cytokine and LPS production, increased gut permeability enabling bacterial endotoxin translocation, and reduced beneficial gut metabolites. Ultimately, systemic inflammation induces insulin resistance and contributes to chronic hyperglycemia and development of complications.

#### 4.4.1 Increased gut permeability

Patients with T2DM have increased intestinal permeability compared to age, sex and BMI matched controls (102). This results in translocation of gut microbes and their products into the bloodstream, in turn causing metabolic endotoxaemia and increased systemic inflammation. This is supported by elevated blood levels of bacterial cell wall products and circulating intestinal bacteria in individuals with pre-diabetes (103) and T2DM (51).

Gut bacterial dysbiosis increases gut permeability via three mechanisms: alterations in expression, canalization and distribution of tight junction proteins; overactivation of the endocannabinoid system; and altered production of beneficial gut metabolites including SCFA and bile acids.

##### 4.4.1.1 Alterations in tight junction proteins

The intestinal lining composed of epithelial cells assisted by tight junctions (TJ), acts as a physical barrier against microorganisms and antigens. TJ controls intestinal permeability (104). In T2DM, reduction in beneficial microbes *Bacteroides*, *Bifidobacterium*, *Faecalibacterium*, *Roseburia* and *Akkermansia*, leads to decreased gene expression and therefore reduced localization, production, and distribution of TJ proteins. This results in increased gut permeability.

Mouse studies show that pre-treatment with *Bifidobacterium* (86), *Bacteroides vulgatus, Bacteroides dorei* (105) or *Prevotella histicola* (97), upregulates TJ genes leading to reduced intestinal permeability and inflammation. *Bacteroides fragilis* (106–108), *Bacteroides facies* (109), *Bifidobacterium bifidum* (110), *Bifidobacterium adolescentis* (111) and *Bifidobacterium longum* (112) have also been found to increase TJ proteins.

*Faecalibacterium prausnitzii* and *Roseburia intestinalis* reduce gut permeability by production of butyrate and upregulation of TJ proteins (89, 109). Butyrate is essential for colonic epithelial cells, offering anti-inflammatory properties and protecting against pathogens (30). In db/db mice, *Faecalibacterium prausnitzii* also produces microbial anti-inflammatory molecule, increasing TJ expression and restoring the damaged intestinal barrier (113).

*Akkermansia muciniphila* decreases gut permeability by promoting TJ protein expression via its outer membrane protein Amuc_1100. Additionally, it improves intestinal TJ via AMPK activation in the epithelium (114) and modulation of the endocannabinoid system (115).

Less understood are the bacteria Rumincoccaeceae and *Blautia* which may be associated with increased gut permeability (116). Further studies are needed to confirm these findings and understand their mechanisms.

##### 4.4.1.2 Endocannabinoid system

There is growing evidence that the endocannabinoid system regulates intestinal inflammation and mucosal barrier permeability, thus influencing T2DM pathophysiology.

The endocannabinoid system, historically associated with cognitive and emotional processes, also regulates intestinal inflammation. The two main endocannabinoids are anadamide (AEA) and 2 arachidonylglycerol (2-AG). They act primarily through cannabinoid receptors CB1R and CB2R. CB1R is expressed in gastrointestinal epithelial cells and myenteric and submucosal plexuses while CB2R may be found on enteric neurons (117).

Overactivation of CB1R via AEA and 2-AG leads to increased gut permeability (117). In T2DM mice models, CB1R antagonists were shown to decrease gut permeability by reducing inflammation and alterations in TJ proteins (118). *Akkermansia muciniphila* antagonises CB1R through its outer membrane protein Amuc_1100, reducing gut permeability, LPS levels and systemic inflammation (115). *Bacteroides fragilis* also affects epithelial barrier permeability through the endocannabinoid system (119).

Oxidative stress, inflammation, and insulin secretion contribute to T2DM and its complications. Although unrelated to gut permeability, CB2R activation decreases inflammation and oxidative stress and promotes pancreatic insulin secretion via calcium signal regulation (120). This suggests potential benefits of CB2R agonists in T2DM management.

#### 4.4.2 Alteration to the gut metabolites

The gut microbiota acts as a metabolic organ and facilitates nutrient and energy harvesting from food. It produces metabolites that regulate host metabolism including SCFA and bile acids which maintain the intestinal barrier (4). Alterations in the gut microbiota is thus associated with alteration to the gut metabolites which in turn contributes to T2DM and its complications.

##### 4.4.2.1 Alteration to short chain fatty acids

SCFAs are produced by gut microbiota from non-digestible carbohydrates. They provide energy to colonocytes, reduce inflammation and regulate satiety (121). The most common SCFAs are acetate, propionate and butyrate, and are predominantly produced by anaerobic Bacteroidetes and Firmicutes phyla.

SCFAs have multiple beneficial effects such as maintaining gut permeability, modulating host metabolism and anti-inflammatory effects. Reduced levels of SCFA-producing bacteria including *Bacteroides*, *Bifidobacterium*, *Faecalibacterium*, *Prevotella* and *Akkermansia*. are associated with T2DM. This is reflected by the reduced acetate (38), propinionate (38, 98), butyrate (38, 98) and other SCFA (38, 51) concentrations in T2DM fecal samples. Functional analysis of gut microbiota showed reduced SCFA-producing pathways in T2DM compared to controls (61).

Individuals with T2DM related complications had lower SCFA fecal concentrations than those without complications (38). Increased dysbiosis severity and reduced production of SCFA may contribute to the development and progression of T2DM complications.

###### 4.4.2.1.1 Alteration to SCFA resulting in decreased gut barrier integrity

SCFA help to maintain gut barrier integrity through a number of mechanisms. This includes promoting epithelial growth and innate responses to microbes, providing energy to intestinal epithelial cells via beta-oxidation in the mitochondrial tricarboxylic acid cycle and maintaining an anaerobic gut environment hostile to opportunistic aerobic pathogens (122). SCFA also stabilize transcription factors that protect the barrier and activate genes for TJ proteins thus preventing bacterial and LPS translocation and systemic inflammation (89). Lower SCFA concentrations in T2DM may therefore to altered microbiota diversity and increased intestinal permeability, predisposing to insulin resistance through metabolic endotoxaemia.

###### 4.4.2.1.2 Alteration to SCFA resulting in altered glucose and lipid metabolism

SCFA influence glucose and appetite regulation. In human *in vivo* studies, rectal infusions of SCFA mixtures led to a rise in plasma peptides YY (123–125) and glucagon peptide-1 (GLP-1) (123). This resulted in appetite control, increased insulin sensitivity and increased pancreatic beta cell concentrations (4, 126). SCFA also modulate glucose and lipid metabolism. Propionate suppresses hepatic gluconeogenesis, while acetate and butyrate reduce lipogenesis and increase leptin secretion (122). In mouse models, SCFA increase food intake via parasympathetic activity and support glucose stimulated insulin secretion (127). Reduced levels of SCFA may therefore lead to poor appetite control, hyperglycemia, hyperlipidemia and insulin resistance.

###### 4.4.2.1.3 Alteration to SCFA results in increased inflammation

SCFA exhibit anti-inflammatory properties. Butyrate inhibits NF-kB activation, reducing pro-inflammatory cytokines like TNF-α, IL-6, IL-2, IL-8 and promotes IL-10 production via GPR109A, maintaining a balance between pro and anti-inflammatory T cells (128). Lower SCFA levels may contribute to chronic inflammatory state and insulin resistance in T2DM.

###### 4.4.2.1.4 Alteration to SCFA negatively disrupting the gut environment

Butyrate producing bacteria compete with gram-negative bacteria, maintaining microflora balance and inhibit pathogenic strains. They also maintain an anaerobic environment by enhancing coloncyte oxygen consumption and stabilizing hypoxia inducible factor (122). Depletion of butyrate producing bacteria can lead an increase in opportunistic pathogens like *Fusobacterium*, which releases harmful by-products perpetuating the inflammatory cycle (129).

##### 4.4.2.2 Alteration to bile acids

Bile acids, known for their role in digestion of dietary fats, have recently gained attention due to their possible influence on metabolic processes, particularly in the context of T2DM. Primary bile acids (PBAs), cholic acid (CA) and chenodeoxycholic acid (CDCA) are synthesized from cholesterol in hepatocytes and released into the duodenum. They are then uncoupled by bile saline hydrolysase before being converted into more hydrophobic secondary bile acids (SBAs) through bile acid deconjugation and the rate limiting 7α-dehydroxylase enzyme. *Bacteroides* and *Enterococcus* are involved in the initial deconjugation, while *Bifidobacterium*, *Lactobacillus* and *Enterococcus* utilize bile saline hydrolase. Meanwhile, selected bacteria from the Lachnospiraceae and Ruminococcaceae family perform the subsequent 7α-dehydroxylase conversion of CA and CDCA to generate the SBAs deoxycholic acid (DCA) and lithocholic acid (LCA) respectively (130). The abundance of these bacteria are described in **Table 8**.

**Table 8:** Abundance of secondary bile acid producing bacteria in type 2 diabetes.

	Increased	Decreased	No significant difference
Ruminococceae		(15, 21, 22, 46)	↑ (11) ↓ (19, 40)
Lachnospiraceae	(18, 32)	(46)	↑ (19) ↓ (11) Equivocal (30)
*Clostridium*	(32)	(11, 22, 35)	↑ (55) ↓ (10, 14, 47, 61)

Studies with no significant differences are reported as trends. ↑ - increase, ↓ - decrease.

Interestingly, the profiles of bile acids in patients with T2DM vary across different studies. Some studies indicate higher levels of total bile acids, PBA and SBA, among individuals with T2DM (131, 132). In contrast, other studies have found no significant differences in total serum bile acid levels between T2DM patients and controls (133). Nonetheless, the majority of these studies do suggest a relationship between increased insulin resistance and higher total bile acids (132, 133), highlighting the therapeutic potential of targeting bile acids in T2DM. Alterations in bile acids have been associated with complications of T2DM including cardiovascular disease (134) and diabetic kidney disease (135).

###### 4.4.2.2.1 Alteration of bile acids resulting in altered glucose metabolism

Bile acids regulate glucose homeostasis through the Farnesoid X receptor (FXR) and Takeda-G-protein-receptor 5 (TGR5) (136). PBAs preferentially activate FXR, while SBAs favor TGR5. Activation of TGR5 appears to have a beneficial effect on glucose metabolism by stimulating release of GLP-1 from enteroendocrine cells, which enhances insulin secretion, slows gastric emptying and reduces appetite (137). Interestingly, both deactivation and activation of FXR have been linked to positive effects on glycemic regulation. For example, intestinal FXR activation has been associated with reduced hepatic gluconeogenesis (138, 139) and contribute to glucagon fasting-induced hepatic gluconeogenesis (140). FXR deficiency has been linked to increased GLP-1 plasma concentrations (138, 141). Nonetheless, hepatic FXR deficiency in mice has been shown to increase gluconeogenesis, worsening glucose intolerance and insulin resistance (142). This FXR paradox highlights the complexity of FXR signaling, and suggests that the role of FXR in metabolic dysfunction may differ between the liver and intestine (143).

The systematic effects of various secondary bile acids on glycemic control have been demonstrated in both humans and animal models. For example, administration of ursodeoxycholic acid (UDCA) has been shown to improve post-prandial glucose levels and GLP-1 secretion (144), reduce metabolic syndrome (145) and increase the survival rate of pancreatic beta cells (146, 147). Additionally, intrajejunal and rectal taurocholic acid led to decreased blood glucose levels and the release of satiety hormones GLP-1 and Peptide YY (148, 149). Meanwhile, metformin, a drug commonly prescribed for T2DM, has been suggested to modulate primary and secondary bile acid levels and alter the expression of their receptors, thereby enhancing insulin sensitivity (150).

Specifically, among the taxa that differ significantly in individuals with T2DM, *Lactobacillus* and *Bifidobacterium* have been suggested to play a role in modulating bile acids and improving glycemic control. In a recent randomized control trial, a probiotic product containing *Lactobacillus casei*, *Lactobacillus plantarum*, *Lactobacillus rhamnosus*, *Bifidobacterium animalis subsp. lactis M8* and *Bifidobacterium animalis subsp. lactis V9.* led to reductions in HbA1c and fasting blood glucose levels, along with increased insulin secretion. Faecal metabolite analysis demonstrated an increase in both CDCA and hyodeoxycholic, a component of hyoholic acid shown to upregulate GLP-1 secretion via TGR5 (139). The study suggested that specific bile acids may activate various receptors, which in turn promotes GLP-1 secretion, thereby reducing blood glucose levels (151). Collectively, these findings highlight the potential therapeutic value of bile acids in T2DM.

###### 4.4.2.2.2 Alteration to bile acids affecting gut barrier integrity

Alterations in bile acid profiles affect intestinal permeability through regulation of TJ proteins. In murine models, DCA reduces TJ protein Zona-Occludens-1, thereby increasing gut permeability (152). Primary biliary acids CDCA and CA, and secondary biliary acids DCA, increase epithelial permeability through phosphorylation of occludin in intestinal Caco cells (153). At high concentrations DCA is cytotoxic to intestinal stem cells and goblet cells, thereby impairing gut permeability (154). Conversely, LCA reduces intestinal permeability by ameliorating TNF-α induced disruption of TJ proteins (155). In murine models, an increase in LCA and DCA was associated with increased colon expression of TGR5 and TJ proteins, thereby improving gut-barrier integrity (156). Human studies demonstrate that elevated levels of LCA and DCA have anti-inflammatory properties within the colon (157). Bile acids have both beneficial and detrimental effects on intestinal permeability, and further studies are required to understand their specific impacts.

###### 4.4.2.2.3 Alteration in bile acids resulting in systemic inflammation

Bile acids have been shown to inhibit the induction of pro-inflammatory genes and the production of inflammatory cytokines by macrophages via FXR and TGFR-5 receptors (158). In mice models, the production of secondary bile acids, such as LCA and UDCA, ameliorated colitis and reduced the production of proinflammatory cytokines TNF- α, IL-17A and IL-6 (156). Alteration in bile acids can thus lead to decreased anti-inflammatory effects and contribute as well as exacerbate the chronic low-grade inflammatory state in T2DM.

In summary, bile acids play a role in modulating intestinal permeability, systemic inflammation, and glucose homeostasis, thereby contributing to the pathogenesis of T2DM. While bile acids represent a promising therapeutic target, the precise abundance of various bile acids in T2DM and their effects on different receptors, particularly FXR, remain unclear. Further studies are needed to confirm these alterations and clarify the specific interactions involved.

##### 4.4.2.3 Increased systemic inflammation

T2DM is associated with chronic low-grade systemic inflammation caused by metabolic endotoxaemia and cytokine stimulation by microbes leading to oxidative stress, macrophage activity and insulin resistance. Insulin resistance occurs due to activation of the inflammatory cascade, subsequent activation of serine kinases, insulin receptor substrate serine phosphorylation and consequent insulin signaling inhibition causing cellular insulin resistance (159).

###### 4.4.2.3.1 Metabolic endotoxaemia

In T2DM, metabolic endoxaemia occurs due to increased production of toxic bacterial components and increased gut permeability enabling translocation of these products into the systemic circulation.

####### 4.4.2.3.1.1 Lipopolysaccharide

Gram-negative bacteria, such as *Fusobacterium* and *Escherichia-Shigella*, produce LPS an endotoxin that activates immune responses by binding to pattern recognition receptors such as toll-like receptor 4 (TLR4), NLRP3 inflammasome and NOD-like receptors which are expressed on the surfaces of antigen presenting cells. This leads to release of pro-inflammatory cytokines IL-1, IL-7, TNF-α release (121) and insulin resistance via inhibition of insulin signaling (159). Gut dysbiosis in T2DM increases LPS synthesis (46, 57) with higher plasma levels of LPS (15, 51) and TLR4 receptor activation (15) observed.

####### 4.4.2.3.1.2 Decreased intestinal alkaline phosphatase due to gut dysbiosis contributes to metabolic endotoxaemia in T2DM

IAP is an enzyme which mitigates intestinal inflammation through detoxification of pathogen toxins and regulation of gut microbes (160). It de-phosphorylates LPS, reducing its toxicity and lowering systemic inflammation (161). In mice, IAP was shown to reverse metabolic endotoxaemia (162). Very low levels of fecal IAP have been reported in T2DM patients (163). *Bifidobacterium* species, *Faecalibacterium prausnitzii, Roseburia* species and other butyrate producing bacteria modulate IAP activity (164). A decrease in these anti-inflammatory, butyrate producing bacteria may contribute to chronic systemic inflammation in T2DM.

###### 4.4.2.3.2 Cytokine modulation

T2DM is associated with elevated pro-inflammatory cytokines. Bacterial taxa such as *Escherichia-Shigella* and *Fusobacterium* are increased in T2DM and correlate with higher levels of pro-inflammatory cytokines like IL-17, TNF-α and IL-6 (165).

Conversely, beneficial microbes *Roseburia intestinalis* (166), *Prevotella histicola(* 97 *)*, *Faecalibacterium prausnitzii (* 167 *), Bifidobacterium longum (* 167 *), Bacteroides fragilis* (87, 168)*, Akkermansia muciniphila(* 169 *)*, *Lactobacillus paracasei* (63) and *Lactobacillus fermentum* (64) promote anti-inflammatory cytokine IL-10 production and suppress pro-inflammatory cytokines (87, 92, 166, 167, 170, 171). Butyrate producing bacteria such as *Roseburia*, *Faecalibacterium* and *Subdoligranulum* also decreases pro-inflammatory cytokine production by inhibiting NF-kB, a major transcription factor essential for inflammatory responses (128).

##### 4.4.2.4 Preferential growth of pathogenic microbiota

Pathogenic bacteria including *Enterococcus* and *Escherichia-Shigella* may outcompete beneficial bacteria, such as *Faecalibacterium*, *Roseburia* and *Bifidobacterium*, perpetuating negative effects on gut health and inflammation.

## 5 Limitations

This systematic review has several limitations. The significant variation in methodology across various human observational studies made it difficult to draw definitive conclusions. Differences in inclusion and exclusion criteria, and varied methods for controlling factors such as age, BMI, diet and medication, affected bacterial abundances and hindered efforts for consistent comparisons. Furthermore, few studies provided raw data on bacterial abundances or reported non-significant bacterial abundances, complicating quantitative data pooling for any specific bacteria.

Most studies did not account for the effects of metformin and other oral anti-hyperglycemic agents, which are known to alter certain bacterial abundances. This review could not control for their use, highlighting the need for future large-scale studies to at least account for, if not control, the effects of these diabetes medications.

Majority of the studies utilized 16s RNA gene sequencing, with few studies utilizing metagenomic sequencing. This meant that it was rare to identify microbes at species or strain levels and may account for some discrepancies at the genus level. Moreover, few studies examined functional alterations in T2DM and correlated it to individual bacterial taxa. Therefore, only associations but not causations between taxa and T2DM could be determined. Future research should assess the functional potential of the gut microbiome in individuals with T2DM.

Finally, the pathogenesis, perpetuation and management of T2DM is multifactorial and various clinical factors including genetics, other comorbidities, adherence to therapies and presence of complications all play a critical role. Future studies should measure these factors, and consider their interplay with gut microbiota in T2DM.

## 6 Conclusion

This systematic review demonstrates that T2DM is strongly associated with gut dysbiosis, as evidenced by differential microbial abundances, altered F/B ratio and changed diversity indices. Through increased gut permeability, decreased SCFA production and modulation of inflammatory cytokines, gut dysbiosis leads to increased systemic inflammation and disrupted glucose homeostasis.

Among these microbes, *Escherichia-Shigella* is consistently associated with T2DM, while *Faecalibacterium*, in particular *Faecalibacterium prausnitzii* appears to offer a protective effect against T2DM. However, the heterogenicity and observational nature of these studies hinder establishment of causative relationships. Future research should control for factors such as age, diet and medication use, and incorporate functional analysis of these gut microbes.

## Data Availability

The original contributions presented in the study are included in the article/**Supplementary Material.** Further inquiries can be directed to the corresponding author.
