# Supplementary material for: A systematic review on gut microbiota in type 2 diabetes mellitus
Source: Front Endocrinol (Lausanne). 2025 Jan 17;15:1486793. doi: 10.3389/fendo.2024.1486793 (PMC11782031; doi:10.3389/fendo.2024.1486793)
Supplement: Supplementary file 2 [file Table2.docx]

**Supplementary 2: Abundance of Bifidobacterium, Roseburia, Prevotella and Bacteroides species**

| **Species** | **Increased** | **Decreased** | **No change** |
| --- | --- | --- | --- |
| *Bifidobacterium bifidum* | ^1^ |  |  |
| *Bifidobacterium longum* |  | ^2^ | ↓^3,4^  ^4,5^ |
| *Bifidobacterium adolescentis* |  | ^2,3^ |  |
| *Bifidobacterium spp.* | **^6^** |  | **^7^** |
| *Bifidobacterium animalis* | ^8^ |  |  |
| *Bifidobacterium infantis* |  |  | ↓^3^ |
| *Bifidobacterium breve* |  |  | ^3^ |
| *Roseburia intestinalis* |  | ^1,9^ | ↑^10^ |
| *Roseburia inulinivorans* |  | ^1,9^ | ↑^11^,↓^5,10^ |
| *Roseburia facies* |  | ^12^ |  |
| *Roseburia hominis* |  | ^12,13^ |  |
| *Roseburia sp.* |  | ^14^ |  |
| *Roseburia 272* |  | ^14^ |  |
| *Bacteroides fragilis* | ^15^ |  | ↑^16^, ↓^4,17^ |
| *Bacteroides vulgatus* |  |  | ↓ ^18,19^  ^20^ |
| *Bacteroides theatiotaomicron* |  |  | ↓ ^19^ |
| *Bacteroides uniformis* |  | ^21^ |  |
| *Bacteroides Intestinalis* | ^9^ | ^14^ | ↓^10^ |
| *Bacteroides caccae* | ^9,13^ |  |  |
| *Bacteroides plebius* | ^12^ |  |  |
| *Bacteroides spp.* | ^22^ | ^6^ |  |
| *Bacteroides sp 20_3* | ^9^ |  | ↓^10^ |
| *Bacteroides finegoldii* | ^13^ |  |  |
| *Bacteroides stercoris* |  | ^21^ |  |
| *Prevotella copri* | ^13^ | ^23,24^ | ↓^5^ |
| *Unclassified Prevotella* |  | ^25^ |  |

1. Wang X, Xu X, Xia Y. Further analysis reveals new gut microbiome markers of type 2 diabetes mellitus. *Antonie Van Leeuwenhoek*. 2017;110(3):445-453.

2. Demirci M, Taner Z, Keskin FE, et al. Similar bacterial signatures in the gut microbiota of type 1 and type 2 diabetes patients and its association with G protein-coupled receptor 41 and 43 gene expression. *Journal of Diabetes and Metabolic Disorders*. December 2022;21(2):1359-1368. doi:<https://dx.doi.org/10.1007/s40200-022-01068-2>

3. Lê KA, Li Y, Xu X, et al. Alterations in fecal Lactobacillus and Bifidobacterium species in type 2 diabetic patients in Southern China population. *Frontiers in Physiology*. 2012;3:496. doi:10.3389/fphys.2012.00496

4. Navab-Moghadam F, Sedighi M, Khamseh ME, et al. The association of type II diabetes with gut microbiota composition. *Microbial Pathogenesis*. September 2017;110:630-636. doi:<https://dx.doi.org/10.1016/j.micpath.2017.07.034>

5. Talukdar R, Sarkar P, Jakkampudi A, et al. The gut microbiome in pancreatogenic diabetes differs from that of Type 1 and Type 2 diabetes. *Scientific reports*. 26 May 2021;11(1):10978. doi:<https://dx.doi.org/10.1038/s41598-021-90024-w>

6. Adachi K, Sugiyama T, Yamaguchi Y, et al. Gut microbiota disorders cause type 2 diabetes mellitus and homeostatic disturbances in gut-related metabolism in Japanese subjects. *J Clin Biochem Nutr*. May 2019;64(3):231-238. doi:10.3164/jcbn.18-101

7. Fassatoui M, Lopez-Siles M, Diaz-Rizzolo DA, et al. Gut microbiota imbalances in Tunisian participants with type 1 and type 2 diabetes mellitus. *Bioscience Reports*. 2019;39(6) Bsr20182348. doi:<https://dx.doi.org/10.1042/BSR20182348>

8. Wang X, Xu X, Xia Y. Further analysis reveals new gut microbiome markers of type 2 diabetes mellitus. *Antonie van Leeuwenhoek, International Journal of General and Molecular Microbiology*. 01 Mar 2017;110(3):445-453. doi:<https://dx.doi.org/10.1007/s10482-016-0805-3>

9. Qin J, Li Y, Cai Z, et al. A metagenome-wide association study of gut microbiota in type 2 diabetes. *Nature*. Oct 4 2012;490(7418):55-60. doi:10.1038/nature11450

10. Zhang X, Shen D, Fang Z, et al. Human Gut Microbiota Changes Reveal the Progression of Glucose Intolerance. *PLoS ONE*. 27 Aug 2013;8(8) A497. e71108. doi:<https://dx.doi.org/10.1371/journal.pone.0071108>

11. Kwan SY, Sabotta CM, Joon A, et al. Gut Microbiome Alterations Associated with Diabetes in Mexican Americans in South Texas. *mSystems*. 2022;7(3)doi:<https://dx.doi.org/10.1128/msystems.00033-22>

12. Wang TY, Zhang XQ, Chen AL, et al. A comparative study of microbial community and functions of type 2 diabetes mellitus patients with obesity and healthy people. *Applied Microbiology and Biotechnology*. 01 Aug 2020;104(16):7143-7153. doi:<https://dx.doi.org/10.1007/s00253-020-10689-7>

13. Zhong H, Ren H, Lu Y, et al. Distinct gut metagenomics and metaproteomics signatures in prediabetics and treatment-naive type 2 diabetics. *EBioMedicine*. September 2019;47:373-383. doi:<https://dx.doi.org/10.1016/j.ebiom.2019.08.048>

14. Karlsson FH, Tremaroli V, Nookaew I, et al. Gut metagenome in European women with normal, impaired and diabetic glucose control. *Nature*. 2013;498(7452):99-103. doi:<https://dx.doi.org/10.1038/nature12198>

15. Ghaemi F, Fateh A, Sepahy AA, Zangeneh M, Ghanei M, Siadat SD. Intestinal Microbiota Composition in Iranian Diabetic, Pre-diabetic and Healthy Individuals. *Journal of Diabetes and Metabolic Disorders*. December 2020;19(2):1199-1203. doi:<https://dx.doi.org/10.1007/s40200-020-00625-x>

16. Chen PC, Chien YW, Yang SC. The alteration of gut microbiota in newly diagnosed type 2 diabetic patients. *Nutrition*. Jul-Aug 2019;63-64:51-56. doi:10.1016/j.nut.2018.11.019

17. Sato J, Kanazawa A, Ikeda F, et al. Gut dysbiosis and detection of "Live gut bacteria" in blood of Japanese patients with type 2 diabetes. *Diabetes Care*. August 2014;37(8):2343-2350. doi:<https://dx.doi.org/10.2337/dc13-2817>

18. Wu X, Ma C, Han L, et al. Molecular characterisation of the faecal microbiota in patients with type II diabetes. *Current Microbiology*. Jul 2010;61(1):69-78. doi:10.1007/s00284-010-9582-9

19. Remely M, Dworzak S, Hippe B, et al. Abundance and diversity of microbiota in type 2 diabetes and obesity. *Journal of Diabetes & Metabolism*. 2013;4(253):2.

20. Remely M, Hippe B, Zanner J, Aumueller E, Brath H, Haslberger AG. Gut microbiota of obese, type 2 diabetic individuals is enriched in Faecalibacterium prausnitzii, Akkermansia muciniphila and Peptostreptococcus anaerobius after weight loss. *Endocrine, Metabolic and Immune Disorders - Drug Targets*. 2016;16(2):99-106. doi:<https://dx.doi.org/10.2174/1871530316666160831093813>

21. Li L, Li C, Lv M, Hu Q, Guo L, Xiong D. Correlation between alterations of gut microbiota and miR-122-5p expression in patients with type 2 diabetes mellitus. *Annals of Translational Medicine*. November 2020;8(22) 1481. doi:<https://dx.doi.org/10.21037/atm-20-6717>

22. Gradisteanu Pircalabioru G, Chifiriuc MC, Picu A, Petcu LM, Trandafir M, Savu O. Snapshot into the Type-2-Diabetes-Associated Microbiome of a Romanian Cohort. *Int J Mol Sci*. Nov 30 2022;23(23)doi:10.3390/ijms232315023

23. Bhute SS, Suryavanshi MV, Joshi SM, Yajnik CS, Shouche YS, Ghaskadbi SS. Gut microbial diversity assessment of Indian type-2-diabetics reveals alterations in eubacteria, archaea, and eukaryotes. *Frontiers in Microbiology*. 14 Feb 2017;8(FEB) (no pagination)214. doi:<https://dx.doi.org/10.3389/fmicb.2017.00214>

24. Saleem A, Ikram A, Dikareva E, et al. Unique Pakistani gut microbiota highlights population-specific microbiota signatures of type 2 diabetes mellitus. *Gut Microbes*. 2022;14(1) 2142009. doi:<https://dx.doi.org/10.1080/19490976.2022.2142009>

25. Kitten AK, Ryan L, Lee GC, Flores BE, Reveles KR. Gut microbiome differences among Mexican Americans with and without type 2 diabetes mellitus. *PLoS ONE*. May 2021;16(5 May) e0251245. doi:<https://dx.doi.org/10.1371/journal.pone.0251245>
