# Supplementary material for: A systematic review on gut microbiota in type 2 diabetes mellitus
Source: Front Endocrinol (Lausanne). 2025 Jan 17;15:1486793. doi: 10.3389/fendo.2024.1486793 (PMC11782031; doi:10.3389/fendo.2024.1486793)
Supplement: Supplementary file 3 [file Table3.docx]

| **PMID** | **Country** | **Sample Size** | **Age** | **BMI** | **Gender** | **Sequencing** | **Genera Positively associated T2D** | **Genera Negatively associated T2D** |
| --- | --- | --- | --- | --- | --- | --- | --- | --- |
| 2040211 |  | T2D =18  HC =36 | T2D: 56±13  HC: 59±11 | T2D: 30±7  HC: 28± 5 | T2D: 18M  HC: 36M | 16S rRNA Tag-encoded amplicon pyrosequencing | **Class**: Betaproteobacteria | **Phylum**: Firmicutes  **Class**: Clostridia |
| 27943013 | China | 300 | NS | NS | NS | Shotgun metagenomics | **Species**:  *Lactobacillus spp., B. animalis, Streptococcus spp, clostridium ramosum, Dialister micraerophilus* | **Species:**  *R. intestinalis, R. inulinivorans, H. influenzae, H.parainfluenzae* |
| N/A | Austria | T2D =24  LHC = 19  OHC = 14 | T2D: 38±14  LHC: 30±8  OHC :38±14 | T2D: 38± 6  LHC: 22± 2  OHC: 34±4 | NS | 16s rRNA sequencing  qPCR *Clostridium cluster IV,XIVa*,  Bacteroidetes, *Bifidobacterium spp* | **Genus**:  *Lactobacillus, Enterobacteria* |  |
| 27577497 | Austria | T2D =14  LHC =18  OHC =14 | T2D: 58±9  LHC: 26±3  OHC :40±15 | T2D: 40±15  LHC: 21±2  OHC: 34±4 | T2D: 14M 10F  LHC: 3M15F  OHC: 7M7F | qPCR *A.muciniphila*, *A.finegoldii*, *B.thetaiotaomicron*, *B.vulgatus*, F*. prausnitz*ii, *L. acidophilus*, *L. helveticus*, *L.reuteri*, *P. anaerobius* | None | None |
| 20087741 | China | T2D =16  HC =12 | T2D: 48-62  HC: 50-65 | NS | T2D: 10M6F  HC: NS | qPCR *B.vulgatus*, *C.leptum*, *Bifidobacterium* | None | **Genus**: *Bifidobacterium* |
| 223386831 | China | T2D =50  HC =30 | T2D: 60±8  HC: 41±11 | T2D: 25±4  HC: 24±3 | T2D: 23M27F  HC: 17M13F | qPCR *Lactobacillus, L. acidophilus*, *L. bulgaricus*, *L.casei*, *L.rhamnosus*, *B.breve*, *B.longum B.infantis* | **Genus**: *Lactobacillus*  **Species**:*L. bulgaricus*, *L.rhamnosum, L. acidophilus* | **Genus**: *Bifidobacterium*  **Species**: *B. adolescentis* |
| 24013136 | China | T2D =13  HC =44 | T2D: 52 (M)  HC: 55 (M) | T2D:27(M)  HC: 23 (M) | NS | 16s rRNA sequencing | **Genus**: *Eubacterium,*  *Lachnospiraceae groups,Ruminococcus*, *Abiotrophia*, *Sporobacter*, *Peptostreptococcus*, *Subdoligranulum*, *Bacteroides*, *Blautia*, *Clostridales*  **Species**: *Clostridiales sp. SS3/4* | **Genus**: *Bacteroides*, *Clostridiales*, *Haemophilus*, *Megamonas*, *Roseburia*, *Blautia*, *Lachnospiraceae group*  **Species**: *F.prausnitzii*, *H. parainfluenzae*, *A. muciniphila* |
| 28328990 | China | Ugyur  T2D=10  HC=10  Kazak  T2D=10  HC=10 | Ugyur  T2D: 57±16  HC: 44±16  Kazak  T2D: 48±14  HC: 35±9 | Ugyur  T2D: 40±2  HC: 28±9  Kazak  T2D: 23±4  HC: 23±5 | NS | 16s rRNA sequencing | None in common | None in common |
| 32214153 | China | T2D =20  HC =40 | NS | T2D: 27±2  HC: 21±2 | T2D: 11M9F  HC: 7M33F | 16s rRNA sequencing | **Phylum:** Firmicutes  **Genus:** *Dorea***,** *Fusobacterium***,** *Streptococcus* | **Phylum:** Bacteroidetes  **Genus:** *Akkermansia*, *Bifidobacterium*, *Faecalibacterium*, *Parabacteroides* |
| 33313226 | China | T2D=30  HC=30 | T2D: 48±7  HC: 46±8 | T2D: 24±3  HC: 22±2 | T2D: 13M16F  HC: 14M16F | 16s rRNA sequencing | **Order:** Veillonellales, Selenomonadales  **Family:** Selenomonadaceae  **Genus:** *Megamonas* | **Phylum**: Bacteroidetes  **Class**: Bacteroidia  **Order**: Bacteroidales Acidaminococcales,  **Family**: Acidaminococcaceae  **Species**: *B. stercoris*, *B. uniformis*, P*. faecium*, *D. Succinatiphilus* |
| 32623494 | China | T2D=6  HC=6 | T2D: 47±5  HC: 44±6 | T2D: 26±2  HC: 23±5 | T2D: 5M1F  HC: 6F | Shotgun metagenomics | **Order**: Acidaminococcales  **Species**: *B. plebius*, *Phascolartobacterium sp_CAG_207* | **Phylum**: Firmicutes  **Class**: Clostridiales, Veillonellales  **Family**: Lachnospiraceae, Ruminococcaceae  **Genus**: *Blautia*, *Cryptobacterium*  **Species**: *F prausnitzii*, *R. faecis*, *S. variabile*, *D. Succinatiphilus*, *R. hominis, uncultured clostridium* |
| 32064276 | China | T2D=134  HC=37 | T2D: 50-67  HC: 45-58 | T2D:23-28  HC:23-27 | T2D:69M65F  HC: 27M27F | 16s rRNA sequencing | **Phylum**:Actinobacteria, Proteobacteria,  **Genus**: *Eggerthella* *Bifidobacterium*, *Cardiobacterium*, *Corynebacterium*, *Eisenbergiella*, *Enterococcus*, *Escherichia-Shigella,* *Faecalibacterium*, *Fusobacterium*, *Granulicatella*, *Lactobacillus*, *Morganella*, *Phascolarctobacterium* | **Phylum**: Bacteroidetes  **Genus**: *Butyricicoccus*, *Cetobacterium*, *Coprococcus*, *Mobiluncus*, *Oscillibacter*, *Paraprevotella*, *Prevotella*, *Roseburia*, *Sneathia* |
| 33224990 | China | T2D=137  HC=179 | T2D: 18-75  HC: 18-75 | NS | T2D:75M62F  HC: 96M83F | 16s rRNA sequencing | **Phylum**: Actinobacteria Proteobacteria, Verruomicrobia  **Genus**: *Akkermansia*, *Bifidobacterium*, *Enterococcus*,Klebsiella, *Escherichia-Shigella*, *Subdoligranulum* | **Phylum**: Bacteroidetes  **Genus**: *Bacteroides*, *Incertae sedis*, *Blautia*, *Faecalibacterium* |
| 33828910 | China | T2D=60  HC=60 | T2D:49±13  HC: 49±13 | NS | T2D:31M29F  HC: 29M31F | 16s rRNA sequencing | **Class:** Negativicutes  **Genus:** *Finegoldia***,** *Megasphaera* | **Genus:** *Bacteroides* |
| 37025407 | China | T2D=183  HC=74 | T2D: 60  HC: 26 | T2D: 25  HC: 22 | T2D:108M75F  HC:26M48F | 16s rRNA sequencing | **Phylum**: Firmicutes, Actinobacteria, Proteobacteria  **Class**:Gammaproteobacteria  **Order**: Lactobacillales Bifidobacteriales, Enterobacteriales  **Family**:Lachnospiraceae Bifidobacteriaceae, Streptococcaceae, Enterobacteriaceae  **Genus**: *Bifidobacterium*, *Blautia*, *Bacilli*, *Shigella*, *Streptococcus*, Coprococcus | **Phylum**: Bacteroidetes  **Class**: Bacteroidia  **Order**: Bacteroidales, Burkholderiales  **Family**: Prevotellaceae  **Genus**: *Proteus*, *Faecalibacterium* |
| 38222790 | China | Han  T2D=12  HC=8  Dai  T2D=12  HC=10 | Han  T2D:55±12  HC: 51±6  Dai  T2D:56±9  HC: 46±15 | NS | Han  T2D 8M4F  HC 3M5F  Dai  T2D 9M3F  HC 5M5F | 16s rRNA sequencing | None in common | None in common |
| 36466492 | China | T2D=74  HC=76 | T2D: 59±8  HC: 59±8 | T2D: 26±4  HC: 24±3 | T2D:29M35F  HC: 29M37F | 16s rRNA sequencing | **Class**: Erysipelotrichia  **Order**: Enterobacteriales, Erysipelotrichales  **Family**: Enterococcaceae, Erysipelotrichaceae  **Genus**: *Blautia*, *Clostridium sensu stricto1*, *Citrobacter*, *Enterococcus*, *Enterobacter*, *Klebsiella* | **Phylum**: Melainabacteria  **Class**: Bacteroidia  **Order**: Bacteroidales, Pasteurellales  **Family**: Clostridiaceae, Acidaminococcaceae  **Genus**: *Coprococcus*, *Haemophilus*, Roseburia, *Lachnospira*, *Lachnospiraceae group*, *Paraprevotella*, *Phascolarctobacterium*, *Ruminococcus* |
| 23023125 | China | Stage 1: T2D= 71, HC= 74  Stage 2: T2D= 100, HC = 100  Validation: T2D = 11, HC= 12 | NS | NS | NS | Shotgun metagenomics | **Family**: Lachnospiraceae  **Genus**:, *Clostridium*, *Alistipes* *Parabacteroides*, *Subdoligranulum*  **Species**: *B. caccae,* *C. hathewayi*, *C. ramosum*, *C. symbiosum, C. bolteae*, *E. lenta*, *E. coli*, *A. muciniphila*, *Desulfovibro sp 3_1_syn3*, *B. intestinalis*, *Bacteroides sp.20_3* | **Order**: Clostridiales  **Family**: Erysipelotrichaceae  **Genus**: *Eubacterium, Faecalibacterium*  **Species**: *Clostridiales sp SS3/4*, *E. rectale,* F*. prausnitzii*, *R. intestinalis*, *R. inulinivorans* |
| 31591683 | China | T2D=65  HC=35 | 52 (M)  44 (M) | T2D: 25±4  HC: 22±1 | T2D:35M30F  HC: 18M17F | 16s rRNA sequencing | **Phylum:** Proteobacteria  **Genus:** *Coprcoccus***,** *Subdoligranulum***,** *Escherichia-Shigella***,** *unknown Ruminococcaceae***,** *unknown Lachnospiraceae* | **Phylum**: Bacteroidetes  **Genus**: *Bacteroides*, *Prevotella* |
| 31492563 | China | T2D=77  HC=97 | T2D:67±8  HC: 59±9 | T2D:50±3  HC: 24±3 | T2D:33M44F  HC:32M65F | Shotgun metagenomics | **Species**: *M. elsdenii*, *B. caccae*, *B. finegoldii,* *C. intestinalis*, *P. copri*, *F. varium*, *Ruminococcus sp.* | **Species**: *A. muciniphila*, *C. bartletti*, *D. invisus*, *R. hominis* , *Roseburia sp.*, *H. parainfluenza*, *Clostridiales sp.*, *C. hathewayi*, *T*.*sanguinis*, *Ruminococcus sp.,* C. *bacterium VE202-14*, *E. cloacae*, *Coprococcus sp.* |
| 34927535 | China | T2D=44  HC=47 | T2D:58±9  HC: 59±12 | T2D:26±4  HC: 25±3 | T2D:24M20F  HC:28M19F | 16s rRNA sequencing | **Genus**: *Faecalibacterium*, *Prevotella*, *Roseburia* | **Genus:** *Shigella*, *Bifidobacterium*, *Veillonella* |
| 26633628 | Denmark | TN T2D=106  Met T2D= 93 | NS | NS | NS | Shotgun metagenomics | None | **Genus:** *Lactobacillus*, *Roseburia*, *Subdoligranulum*, *Paraprevotella*, *Haemophilus* |
| 33658058 | Denmark  India | Denmark  T2D=241  HC=138 India  T2D=157  HC=137 | Denmark  35-75  India  35-65 | Denmark  20-40  India  16-51 | NS | 16s rRNA sequencing | **Genus:** *Lachnoclostridium*, *CAG-56 (family Lachnospiraceae)* | **Genus:** *Subdoligranulum*, *Butyricicoccus* |
| N/A | Egypt | T2D=79  HC=100 | T2D:59±16  HC: 56±8 | NS | T2D:50M29F  HC:60M40F | PCR primers - Lactobacillus, L*. casei*, *L. reuteri*, *L. acidophilus, L. delbrueckii*, L gasseri, *L. plantarum*, *L. rhamnosus* | **Species:** *L. acidophilus, L.rhamnosus* | **Species:** *L. gaesseri*, *L. reuter*i, *L. plantarum* |
| 23719380 | Europe | T2D=53  HC=43 | 70 | NS | T2D:53F  HC:43F | Shotgun metagenomics | **Species:** *Lactobacillus sp., L. gasseri, S. mutans, C. clostridioforme* | **Family:** Coriobacteriaceae  **Species:** *Roseburia 272***,** *E. eligens*, *B. intestinalis* |
| 32234773 | India | NDM=11  KDM=39  HC=35 | NDM:45±7  KDM:52±7  HC: 37±8 | NDM:28±3  KDM:26±2  HC: 25±3 | NS | 16s rRNA sequencing | **Phylum:** Firmicutes, Proteobacteria | **Phylum:** Bacteroidetes |
| 28261173 | India | NDM=14  KDM=16  HC=19 | NDM:49±6  KDM:51±3  HC: 49±5 | NDM:28±2  KDM:27±4  HC: 26±4 | NS | 16s rRNA sequencing  PCR *Lactobacillus* and Bacteroidetes | **Phylum:** Firmicutes  **Genus:** *Lactobacillus* | **Phylum**:Proteobacteria  **Family**: Ruminococcaceae  **Genus**: Lachnospiraceae OTU  **Species**: *P. copri*, *F. prausnitzii* |
| 34040023 | India | T2D=10  HC=9 | T2D: 49±7  HC: 43±13 | T2D: 20±2  HC: 21±2 | T2D:7M3F  HC:6M3F | 16s rRNA sequencing | None | **Species:** *R. bromii*, *B. fibrisolvens* |
| 27366720 | India | T2D=17  HC=13 | 22-70 | T2D:20-39  HC:20-33 | NS | Metagenomic sequencing | **Genus:** *Escherichia* | **Genus:** *Bifidobacterium* |
| 33520834 | Iran | T2D=18  HC=30 | T2D: 53.3  HC: 51.63  40-60 | NS | NS | qPCR primers- *Akkermansia*,  *Lactobacillus Bacteroides*, E. Coli, *Bifidobacterium*, *Faecalibacterium* | **Genus:** *Lactobacillus*  **Species:** *E. Coli, B. fragilis* | **Genus:** *Bifidobacterium*  **Species*:*** *A. muciniphila, F. prausnitzii* |
| 28912092 | Iran | T2D=18  HC=18 | T2D: 54±8  HC: 52±8 | T2D: 26±3  HC: 24±3 | T2D:7M11F  HC:7M11F | Real-time qPCR - *Lactobacillus*, *Bifiodbacterium*, *Fusobacterium*, *Prevotella* | **Genus:** *Lactobacillus* | **Genus:** *Bifidobacterium* |
| 28739439 | Iran | T2D=18  HC=18 | T2D: 54±8  HC: 52±8 | T2D: 26±3  HC: 24±3 | T2D:7M11F  HC:7M11F | Real-time qPCR- *B. fragilis*, *B. longum*, *F. prausnitzii* |  | **Species:** *F. prausnitzii* |
| 27151248 | Italy | T2D=40  HC=13 | T2D:40-77  HC: 21-40 | T2D: 34±6  HC: 22±2 | T2D:20M20F  HC:4M9F | 16s rRNA sequencing | **Genus:** *Collinsella*, *Ruminococcus*, *Lactobacillus* | **Genus**: *Bacteroides Bifidobacterium*, *Lachnospira*, *Faecalibacterium*, *Oscillospira* |
| 24824547 | Japan | T2D=50  HC=50 | T2D:63±11  HC: 60±13 | T2D:26(M)  HC: 22(M) | T2D:26M24F  HC:26M24F | RT qPCR | **Genus**: *Lactobacillus*  **Species**: *L. reuteri*, *L. plantarum* | **Genus:** *Atpobium*, *Prevotella*  **Species:** *C. coccoides* |
| 29203964 | Japan | T2D=10  HC=12 | T2D:65±8  HC: 62±7 | T2D:30±7  HC: 26±3 | T2D:5M5F  HC:8M4F | 16s rRNA sequencing |  | **Genus:** *Blautia* |
| 35683603 | Japan | T2D=96  HC=923 | T2D:61±11  HC: 51±14 | T2D:25±4  HC: 23±3 | T2D:44M52F  HC:359M564F | 16s rRNA sequencing  RT qPCR | **Genus:** *Bifidobacterium*, *Streptococcus* | **Genus:** *Roseburia*, *Blautia* |
| 31138957 | Japan | T2D=59  HC=59 | T2D:64(M)  58-69  HC: 62 (M)  59-69 | T2D:23(M)  20-26  HC:23(M)  21-24 | T2D:34M25F  HC:34M25F | Terminal restriction fragment length polymorphism technique | **Order**: Lactobacillales  **Species**: *Bifidobacterium spp.* | **Species:** *Bacteroides spp.* |
| 33488518 | Mexico | T2D=48  HC=214 | T2D:51±9  HC: 38±14 | T2D:33±7  HC: 27±5 | T2D:17M31F  HC:49M165F | 16s rRNA sequencing | None | None |
| 34579166 | Netherlands | AS  T2D (Met) =111  T2D (TN)=78  HC= 189  SAS  T2D (Met)=128  T2D (TN)=49  HC=177 | AS  T2D (Met): 59±7  HC: 57±7  T2D (TN): 57 (53-61)  HC:57(52-63)  SAS  T2D (Met): 58(55-65)  HC:45(34-51)  T2D (TN): 58(50-62)  HC:53 (49-59) | AS  T2D (Met): 30±5  HC: 29±5  T2D (TN): 31±7  HC: 29±5  SAS  T2D (Met): 28 (25-31)  HC: 24 (22-27)  T2D (TN): 27(24-30)  HC:26 (24-28) | AS  T2D (Met): 38M73F  T2DTN:41M37F  HC:87M102F  SAS  T2D (Met): 75M53F  T2DTN:23M26F  HC:69M102F | 16s rRNA sequencing | None in common | None in common |
| 32490229 | Nigeria | T2D=20  HC=22 | T2D:70±6  HC: 69±6 | NS | T2D: 6M14F  HC: 14M8F | 16s rRNA sequencing | None | **Family**: Ruminococcaceae, Clostridiaceae  **Genus**: *Clostridium* |
| 32158702 | Nigeria | T2D=98  HC=193 | T2D:60±10  HC: 54±13 | T2D:32±6  HC: 30±6 | NS | 16s rRNA sequencing | **Phylum**: Bacteroidetes, Euryarchaeota  **Genus**: *Prevotella*, *Peptostreptococcus*, *Eubacterium*  **Species**: *D. piger* | **Phylum**: Firmicutes  **Family**: Clostridiaceae, Peptostreptococcaceae  **Genus**:, *Anaerostipes*, *Clostridium*, *Collinsella Ruminococcus*, *Epulopiscium*, *Adlercreutzia*, *Pediococcus*  **Species**: *R. lactaris*, *C. ruminicola*,  *C. paraputrificum*,  *C. butyricum* |
| 37605183 | Norway | T2D=16  HC=48 | T2D:64(M)  54-72  HC: 48(M)  26-50 | T2D:30(M)  27-34  HC: 23(M)  21-25 | T2D: 13M3F  HC: 12M36F | 16s rRNA sequencing | **Class**: Erysipelotrichales  **Family**: Erysipelotrichaceae  **Genus**: *Dorea*, *Turicibacter*  **Species**: *D. formicigenerans*, *D. longicatena*, *T. sanguinis* | **Genus:** *Anerotignum* |
| 31891582 | Pakistan | T2D=40  HC=20 | T2D:38±12  HC: 38±12 | T2D:32±4  HC: 22±3 | NS | 16s rRNA sequencing | **Phylum**: Firmicutes  **Class**: Negativicutes, Coriobacteriia, Clostridia  **Genus**: *Dialister*, *Allisonella*  **Species***: E. coprostanoligenes group* | **Phylum**: Bacteroidetes, Verrucomicrobia, Proteobacteria, Elusimicrobia  **Class**: Bacteroidia Verrucomicrobiae, , Gammaproteobacteria, Elusimicrobia  **Genus**: *Ruminococcus*, *Prevotella*, *Escherichia-Shigella* |
| 36322821 | Pakistan | T2D=46  HC=48 | T2D:52(M)  HC: 49(M) | T2D:28(M)  HC: 25(M) | NS | 16s rRNA sequencing | **Family**: Lactobacillaceae, Coriobacteriaceae, Atopobiaceae, Bifidobacteriaceae, Eggerthellaceae  **Genus**: *Libanicoccus*, *Lactobacillus*, *Collinsella*, *Senegalimassilia*, *Bifidobacterium*, *Slackia*, *Blautia*, *Dorea*  Species*: C. aerofaciens*, *C. bouchesdurhonensis* | **Family:** Prevotellaceae, Ruminococcaceae  **Genus**: *Oribacterium*, *Faecalibacterium*, *Prevotella*, *Dialister*  **Species**: *F. prausnitzii*, *P. Copri*, *Ruminococcaceae UCG 002 bacterium* |
| 29657308 | Poland | T2D=23  HC=22 | T2D:60(M)  57-63  HC: 37(M)  31-48 | T2D:28(M)  25-32  HC: 23(M)  22-25 | T2D:15M8F  HC:7M16F | 16s rRNA sequencing | **Genus**: *Ruminococcus*, *unclassified Enterobacteriaceae*, *unclassified Flavobacteriaceae* | **Phylum**: Bacteroidetes  **Genus**: *Unclassified Clostridiaceae*, *Unclassified Peptostreptococcaceae*, *Unclassified Lachnospiraceae*, *Bacteroides*, *Roseburia* *Anaerostipes* |
| 36499348 | Romania | T2D=105  HC=45 | T2D:63±12  HC: 57±10 | T2D:30±4  HC: 24±2 | T2D:31M74F  HC:15M30F | RT PCR 16s rRNA sequencing | **Phylum**: Bacteroidetes, Proteobacteria  **Family**: Enterobacteriaceae  **Species**: *Bacteroides spp.*, *Fusobacterium spp.* | **Phylum**: Firmicutes, Tenericutes  **Species**: *A. Muciniphila*, *F. prausnitzii*, *Butyricicoccus spp*., *Lactobacilli spp*., *Ruminococcus spp.* |
| 26555712 | Russia | T2D=23  HC=49 | T2D:58±9  HC: 48±14 | T2D:30±6  HC: 25±4 | T2D:10M13F  HC:11M38F | 16s rRNA sequencing | **Genus:** *Blautia* |  |
| 33062716 | Sudan | T2D=24  HC=24 | T2D: 54  HC: 47 | T2D:25  HC:25 | T2D:12M12F  HC: 12M12F | 16s rRNA sequencing | **Phylum**: Actinobacteria  **Genus**: *Catenibacterium*, *Holdemanella*, *Parvimonas, Bifidobacterium*, *Fusobacterium*, *Blautia* | **Phylum**: Elusimicrobia Proteobacteria  **Genus**: *Elusimicrobium, Faecalibacterium*, *Dialister*, *Succinivibrio* |
| 30933725 | Taiwan | T2D=50  HC=50 | T2D:51±12  HC: 52±10 | T2D:27±4  HC: 23±2 | T2D:36M14F  HC:28M22F | 16s rRNA sequencing  qpCR primers: *Bidifobacterium*, *Lactobacillus*, *C. perfringens,* Enterobacteriaceae | **Genus:** *Lactobacillus* | **Species:** *C. coccoides*, *C. leptum* |
| 31147456 | Tunisia | T2D=10  HC=11 | 20-67 | T2D:29±3  HC: 23±4 | T2D:4M6F  HC:3M8F | 16s rRNA PCR - Firmicutes, Bacteroidetes, F*. prausnitzii*, *A. muciniphila*, *Bifidobacterium spp.* |  | **Phylum**: Firmicutes  **Species**: *A. Muciniphila*, *F. prausnitzii* |
| 37669144 | Tunisia | T2D=10  HC=10 | T2D:56±10  HC: 31±7 | T2D:28±3  HC: 23±4 | T2D:4M6F  HC:5M8F | 16s rRNA sequencing | **Phylum:** Bacteroidetes | **Phylum**: Firmicutes, Verrucomicrobia  **Genus**: *Oscillibacter*, *Faecalibacterium*, *Clostridium*, *Blautia*  Species: *A. muciniphila* |
| 36404809 | Turkey | T2D=40  HC=40 | T2D:32±4  HC: 31±5 | T2D:28±4  HC: 21±2 | T2D:20M20F  HC:20M20F | qPCR A. hallii, *A. muciniphila*, *B. adolescentis*, *B. longum*, *C aerofaciens*, *F prausnitzii*, *L. rhamnosus*, *P. distasonis* | **Species:** *A. Muciniphila* | **Species**: *B. longum*, *F. Prausnitzii*, *B. adolescenti*s, *A. hallii* |
| 32541680 | UAE | T2D=25  HC=25 | T2D:64(M)  49-67  HC: 26(M)  21-26 | T2D:25(M)  23-26  HC: 28(M)  27-34 | T2D:9M16F  HC:3M22F | 16s rRNA sequencing | **Phylum:** Lentisphaerae  **Order:** Victivallales  **Family:** Victivallaceae  **Genus:** *Mogibacterium Phascolarctobacterium***,** *Acidaminococcus,*  *Unclassified Victivallaceae* | **Genus:** *Odoribacter*, *Lactococcus``* |
| 26756039 | USA | T2D=14  HC=15 | T2D:62±10  HC: 55±14 | T2D:32±7  HC: 29±5 | T2D:6M8F  HC: 5M10F | 16s rRNA sequencing | **Genus:** *Collinsella*, *Enterobacteriaceae group* |  |
| 33983982 | USA | T2D=14  HC=23 | T2D:68(M)  59-72  HC: 55(M)  38-61 | T2D:30(M)  26-36  HC: 28(M)  27-31 | T2D:5M9F  HC:5M18F | 16s rRNA sequencing | **Genus:** Unclassified Lachnospiraceae  **Species**: *R. torques*, *Unclassified Clostridium*, *Unclassified Ruminococcus*, *Unclassified Blautia* | **Species*:*** *Unclassified Prevotella*, *Unclassified Streptococcus* |
| 35477306 | USA | T2D=75  HC=141 | T2D:57(M)  34-81  HC: 54(M)  19-89 | T2D:32(M)  21-50  HC: 30(M)  19-49 | T2D:24M51F  HC:38M103F | 16s rRNA sequencing  Shotgun metagenomic sequencing | **Phylum:** Proteobacteria  **Class:** Gammaproteobacteria  **Order:** Enterobacterales  **Family:** Enterobacteriaceae  **Genus:** *Escherichia-Shigella* | **Family:** Clostridiaceae_1**,** peptostreptococcae  **Genus:** *Clostridium sensu stricto 1***,** *Romboutsia***,**  **Species:** *R. timonensis***,** *S. termitidis***,** *C. saudiense* |
| 34161346 | USA | UDT2D = 154  T2D=307  HC=307 | UDT2D: 69±3  T2D:70±3  HC: 69±3 | UDT2D: 28±5  T2D:30±5  HC: 27±5 | UDT2D:82M71F  T2D:167M140F  HC:314M421F | 16s rRNA sequencing | **Phylum:** Lentisphaerae  **Genus:** *Escherichia-Shigella*, *Lachnospiraceae uncultured* | **Phylum**: Firmicutes Actinobacteria, Synergistetes  **Genus**: *Clostridium sensu stricto 1*, *Lachnospira*,uncultured Peptostreptococcaceae |
| 33426245 | Vietnam | T2D=7  HC=7 | T2D:52-70  HC:24-43 | T2D:30-32  HC:18-22 | T2D:7F  HC:7F | 16s rRNA sequencing |  | **Phylum:** Firmicutes  **Class:** Clostridia |

Summary

NS: Not Stated, HC: Healthy Control, T2D: Type 2 Diabetes Mellitus, LHC: Lean Healthy Control, OHC: Obese Healthy Control, (M): Median, TN: Treatment Naive, Met: Metformin, NDM: New Diabetes Mellitus, KDM: Known Diabetes Mellitus, AS: African Surminamese, SAS: South Asian Surminamese, UD: Undiagnosed Values were rounded up or down to whole numbers.
